# When metal-catalyzed C–H functionalization meets visible-light photocatalysis

**DOI:** 10.3762/bjoc.16.147

**Published:** 2020-07-21

**Authors:** Lucas Guillemard, Joanna Wencel-Delord

**Affiliations:** 1 Laboratoire d’Innovation Moléculaire et Applications (UMR CNRS 7042), Université de Strasbourg/Université de Haute-Alsace, ECPM, 25 rue Becquerel, 67087, Strasbourg, France

**Keywords:** C–H activation, C–H functionalization, dual catalysis, photoredox catalysis, radical chemistry, sustainable synthesis, synergistic catalysis

## Abstract

While aiming at sustainable organic synthesis, over the last decade particular attention has been focused on two modern fields, C–H bond activation, and visible-light-induced photocatalysis. Couplings through C–H bond activation involve the use of non-prefunctionalized substrates that are directly converted into more complex molecules, without the need of a previous functionalization, thus considerably reduce waste generation and a number of synthetic steps. In parallel, transformations involving photoredox catalysis promote radical reactions in the absence of radical initiators. They are conducted under particularly mild conditions while using the visible light as a cheap and economic energy source. In this way, these strategies follow the requirements of environment-friendly chemistry. Regarding intrinsic advantages as well as the complementary mode of action of the two catalytic transformations previously introduced, their merging in a synergistic dual catalytic system is extremely appealing. In that perspective, the scope of this review aims to present innovative reactions combining C–H activation and visible-light induced photocatalysis.

## Introduction

In recent years, the field of synthetic chemistry has witnessed a profound evolution. Indeed, due to the growing environmental awareness, organic chemists face nowadays an urgent need of developing much more sustainable and environmentally benign routes to afford molecular complexity from simple precursors, while concomitantly limiting the number of steps and waste generation. Following this general key objective, the landscape of organic synthesis has been clearly changed by the development of ecofriendly methodologies such as metal-catalyzed C–H activations, visible-light-induced photocatalysis, electrosynthesis, enzyme catalysis, and others. Each of these techniques aims at accessing complex molecules while limiting ecological footprint.

Over the last decade, the metal-catalyzed C–H activation established itself as one of the most rapidly expanding domain of organic synthesis [1–14]. Indeed, this approach tends in rendering the classical metal-catalyzed cross-coupling more environmentally friendly, as simple, non-prefunctionalized substrates could be used in the presence of a metallic catalyst, able to insert selectively into one specific C–H bond. Accordingly, not only waste generation was significantly reduced, but also new retrosynthetic connections were envisioned, allowing the conception of more straightforward synthetic protocols [15–20]. Besides, the formation of previously inaccessible molecules could be expected. C–H activation reactions, generally do not change the oxidation state of the metal and are favored in the case of aromatic or vinylic substrates but are clearly more challenging when using aliphatic precursors [21–28].

In parallel, in the 21st century there is a real renaissance for photocatalysis, with great advances achieved mainly in visible-light-mediated reactions [29–43]. Various photosensitizers, initially based on noble metals (such as Ir and Ru polypyridine complexes) [38–39], and more recently, organic compounds (cyanoarenes, xanthenes, thiazines, pyryliums or acridiniums) [40], emerged. These molecules capable of harvesting visible light from simple household bulbs or easily accessible LEDs, opened the door towards the design of numerous electron or energy-transfer-type reactions under mild conditions and in the absence of toxic radical precursors [44–46].

Considering, on one hand, the fundamentally appealing properties of both, metal-catalyzed C–H functionalization reactions and visible-light-induced photocatalysis, and, on the other hand, their complementary reactivities, the combination of these two activation modes could provide new solutions for sustainable organic synthesis.

**Figure 1:**
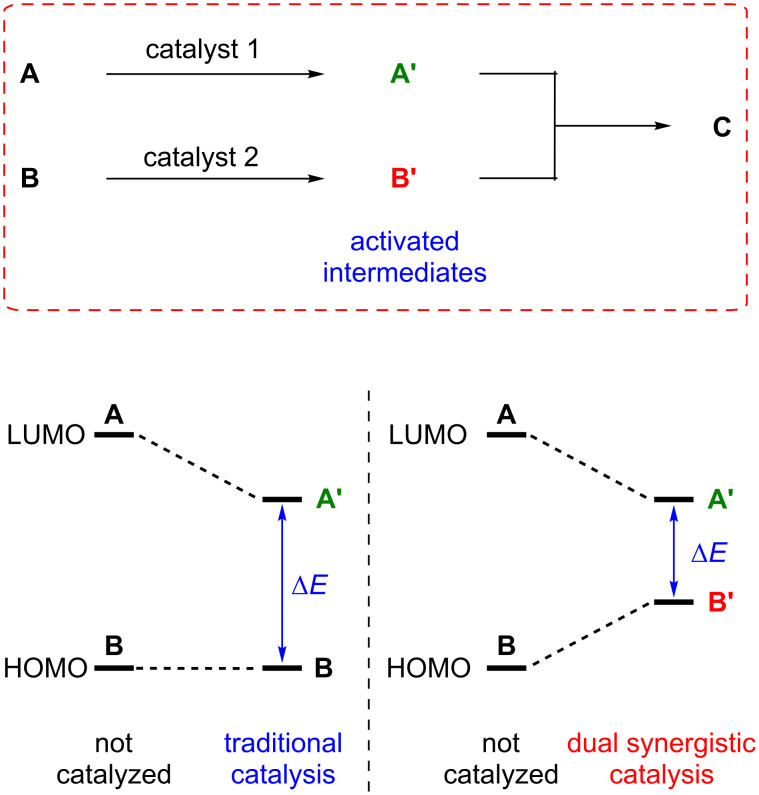
Concept of dual synergistic catalysis.

Indeed, new mutlicatalysis concepts have emerged recently and enabled the achievement of various yet unfeasible reactions [47]. Following this general goal, synergistic catalysis involving two catalysts and two catalytic cycles acting in a cooperative way to create a new bond, turned out to be a powerful strategy. According to a simplified representation, synergistic catalysis involves the concurrent activation of a nucleophile and an electrophile by means of two distinct catalysts. This simultaneous activation then produces two reactive species, one with a higher HOMO and another with a lower LUMO, in comparison to the respective ground-state energy of both substrates (Figure 1) [47]. By judiciously choosing the starting materials, the coupling of the activated intermediates promotes chemical transformations generally unattainable using traditional monocatalysis.

**Figure 2:**
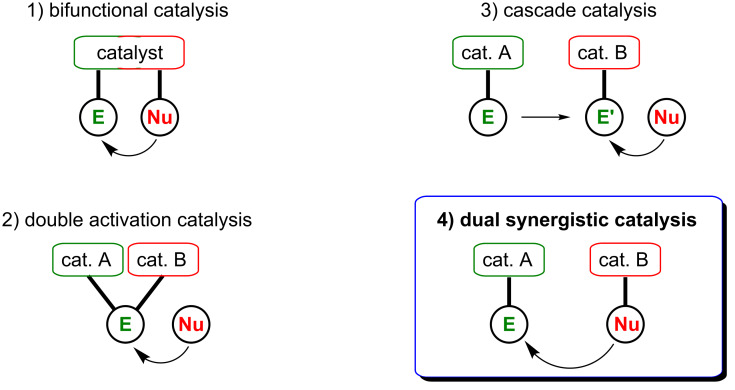
Classification of catalytic systems involving two catalysts.

**Figure 3:**
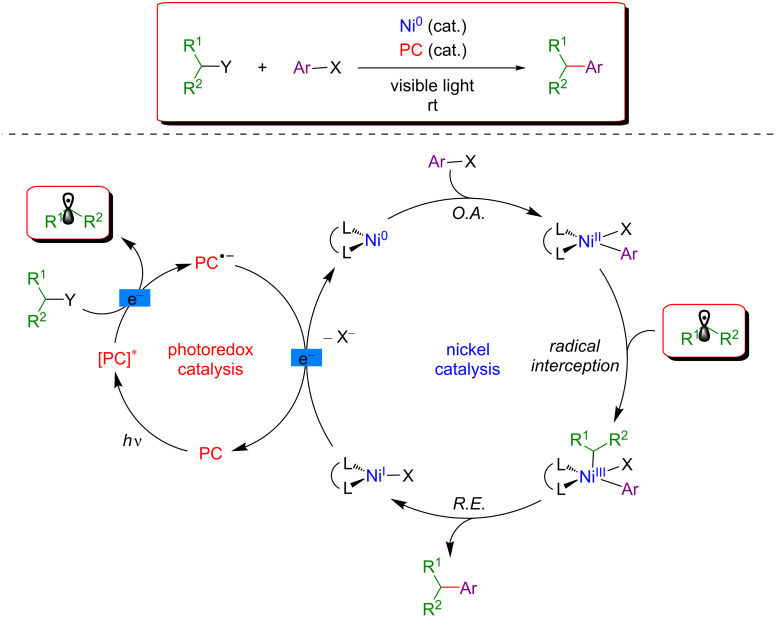
General mechanism for the dual nickel/photoredox catalytic system.

**Figure 4:**
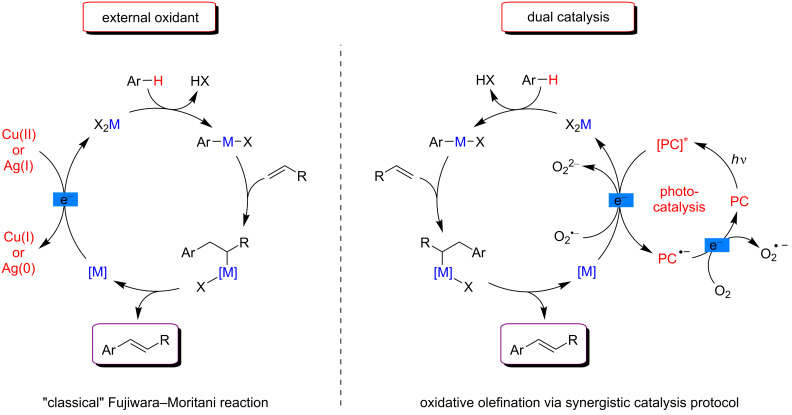
General mechanisms for C–H activation catalysis involving different reoxidation strategies.

However, the potential of multicatalyzed mechanisms goes far beyond the category of synergistic catalysis and the interest in this field fructified in the development of different possible scenarios. First, if both the nucleophile and the electrophile are activated independently by distinct functional groups on the same catalyst, this is referred to as bifunctional catalysis (Figure 2, (1)) [48]. When the two catalysts work in a cooperative way to activate only one substrate, this is classified as double activation catalysis (Figure 2, (2)) [49–50]. Similarly, if both catalysts activate the same coupling partner but in a sequential fashion (the activated substrate leads to an intermediate further activated by the second catalyst), this falls in the category of cascade catalysis (Figure 2, (3)) [51–52]. Finally, the panel of these multicatalyzed reactions is complemented with the above-mentioned synergistic catalysis strategy, i.e., the simultaneous activation of an electrophile and a nucleophile by two distinct catalysts in order to achieve a single chemical transformation (Figure 2, (4)) [47].

Despite great advances achieved in the field of both, C–H activation and visible-light-induced photocatalysis, dual systems merging these two activation modes have remained underdeveloped until recently. In contrast, over the past decade, the design of new strategies combining photoredox catalysis with a second catalytic system has sparked significant interest from the scientific community. Accordingly, numerous unprecedented reactions involving photoredox catalysis in synergy with other activation modes including Brønsted/Lewis acid catalysis, organocatalysis, enzymatic biocatalysis, or electrocatalysis, have been achieved recently [53–58].

In addition, various transformations merging photoredox catalysis with transition-metal catalysis have been disclosed [59–65]. Among these different strategies, visible-light photoredox catalysis combined with nickel catalysis is undoubtedly the most thoroughly investigated approach and consequently, the most widely described in the literature [66–67]. The interest of the scientific community towards this dual catalysis and further developments were pioneered by MacMillan [68] and Molander [69]. The general mechanism of such transformations involving the dual Ni/photoredox catalytic system is presented in Figure 3.

Following the idea of combining C–H activation and photoredox catalysis in multicatalyzed complex systems, several different approaches were designed. The initial examples of merging these two domains concerned the development of “classical” C–H activation reactions, while a photoredox cycle was implemented to reoxidize the metal catalyst, thus obviating the need for a stoichiometric amount of metal-based oxidants, such as silver or copper salts.

In parallel, dual synergistic catalysis has emerged. In this case, C–H activation and photocatalysis were used separately to activate, via fundamentally very different activation modes, two coupling partners. The catalytic cycles were successfully interconnected and such strategies benefit from the cooperative activation of distinct substrates. Interception of the independently formed intermediate furnished the expected coupling products under generally mild reaction conditions. Furthermore, a merge of metal-catalyzed C–H functionalization and photocatalysis was also astutely employed to design modern versions of Sonogashira-type reactions as well as mild heteroaromatic functionalizations. Last but not least, a HAT-type process inducing the generation of alkyl radicals via selective C–H abstraction from the aliphatic precursors may be combined with a Ni-catalyzed process, thus delivering cross-coupled products.

The aim of this review is to present the variety of possibilities in combining transition metal C–H activation and visible-light photoactivation to reach sustainable synthesis. The particular focus is put on the mechanisms of such complex catalytic systems, highlighting the unique reactivity that arises from the original combination of these two activation modes. The advantages of the dual catalytic systems over traditional monocatalyzed approaches is also discussed, thus illustrating their sustainable character.

## Review

### Visible-light-mediated photocatalysis as a sustainable reoxidation strategy

In the context of traditional C–H bond activation, a catalytic amount of a transition metal (generally Pd, Rh and Ru) is frequently used in combination with a stoichiometric amount of an external oxidant (typically Cu or Ag salts). Such additional oxidants are required if transiently produced low-valent metal complexes need to be reoxidized by electron transfer to complete the catalytic cycle and regenerate the active catalytic species. The classical Fujiwara–Moritani reaction promoting the addition of arenes to olefins illustrates a general mechanism for such traditional C–H functionalization (Figure 4, left) [70–72]. The insertion of a metal catalyst X_2_M into an aromatic C–H bond of a substrate (generally facilitated by the presence of a directing group (DG)), delivers a metal–aryl complex. Coordination and subsequent insertion of an alkene into the M–aryl bond then provides the desired coupling product after β-hydride elimination, together with a metal hydride or a low-valent metal complex. Hence, in order to reoxidize the metal catalyst, excess of an external oxidant, such as Cu(II) or Ag(I) salts, was frequently used.

On the other hand, photoredox catalysis has been mainly employed for electron-transfer reactions and, remarkably, few examples have been reported in which the photoredox process modifies the oxidation state of a catalyst [55–56]. Subsequently, C–H activation protocols benefiting from mild photocatalytic reoxidation have spread rapidly [60–62]. In such a case, a photocatalyst (PC) is introduced in the reaction media in order to transfer electrons from the low-valent metal complex formed in situ after reductive elimination of the product (Figure 4, right). In this way, the metalacyclic intermediate is reoxidized while the photosensitizer is reduced, thus completing the C–H activation catalytic cycle. By selecting judiciously a PC with adequate redox potentials, the ground state of the latter could be regenerated by means of a mild and abundant oxidant such as molecular oxygen. The overall process thus allowed the replacement of stoichiometric amounts of external oxidants with a suitable PC.

**Figure 5:**
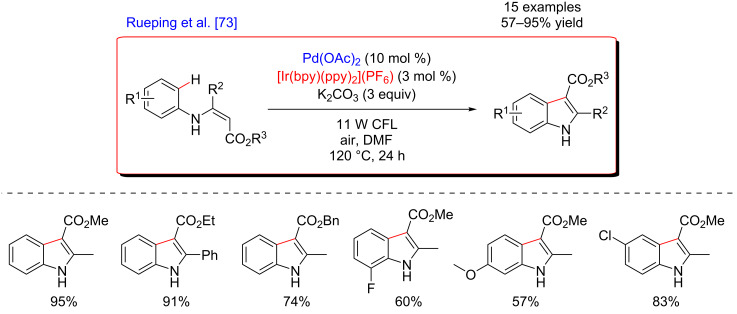
Indole synthesis via dual C–H activation/photoredox catalysis.

Following this general strategy, in 2014, Rueping and co-workers reported the pioneering contribution concerning the synthesis of indoles via an intramolecular C–H/C–H oxidative coupling of *N*-arylenamines under air atmosphere. This procedure was based on dual catalysis involving a double C–H activation system and photoredox catalysis (Figure 5) [73]. The coupling products were generally isolated in good yields, although this procedure required high temperature (120 °C).

**Figure 6:**
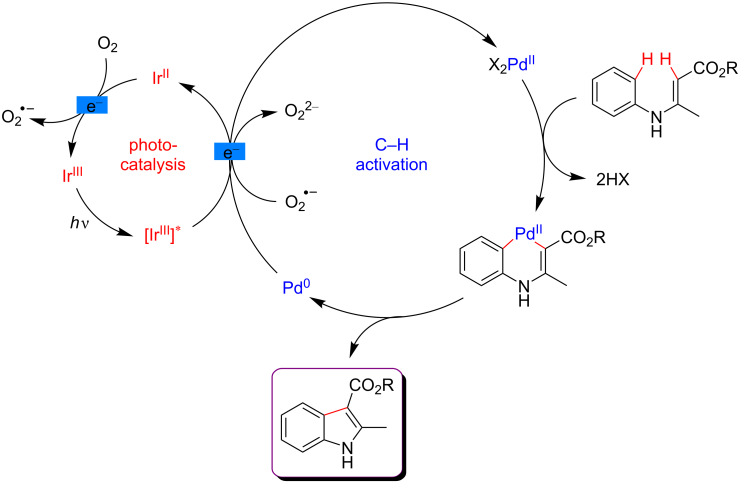
Proposed mechanism for the indole synthesis via dual catalysis.

Remarkably, molecular oxygen was used as the terminal oxidizing agent of the overall C–H functionalization reaction. The mechanistic studies revealed that both, the excited Ir photocatalyst and the superoxide anion generated during the transformation, were able to oxidize the low-valent Pd(0) species resulting from the reductive elimination (Figure 6). Under such dual catalysis protocol, various oxidant-sensitive functional groups were tolerated, thus rendering this methodology well suitable for the synthesis of a variety of indole derivatives.

Subsequently, the panel of oxidative Heck coupling reactions via a photoredox catalytic system combined with C–H activation using Rh [74] or Ru [75] was extended by Rueping’s group.

**Figure 7:**
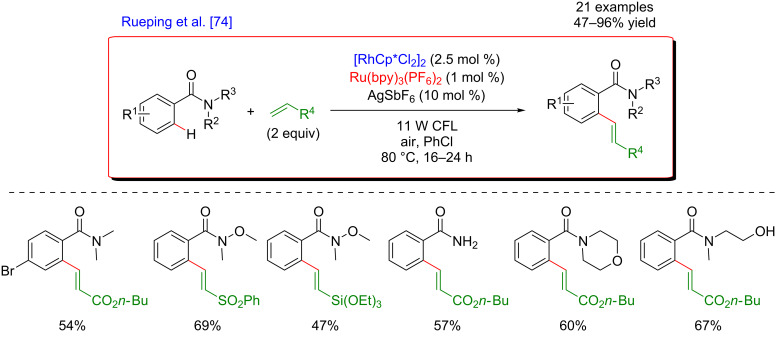
Oxidative Heck reaction on arenes via the dual catalysis.

In 2014, Rueping described the combination of rhodium and photoredox catalysis for the direct C–H *ortho*-olefination of arylamides (Figure 7) [74]. The procedure was performed under air at 80 °C and turned out to be efficient using a low loading of a Ru-based photosensitizer. A broad range of DGs could be installed on the aromatic coupling partner, including frequently used Weinreb amides or functionalized secondary and primary amides. Regarding the scope of olefin substrates, the reaction tolerated numerous functional groups including acrylates, vinyl silanes or sulfones which was interesting from the post-functionalization viewpoint, thus furnishing a large panel of compounds in excellent to good yields.

**Figure 8:**
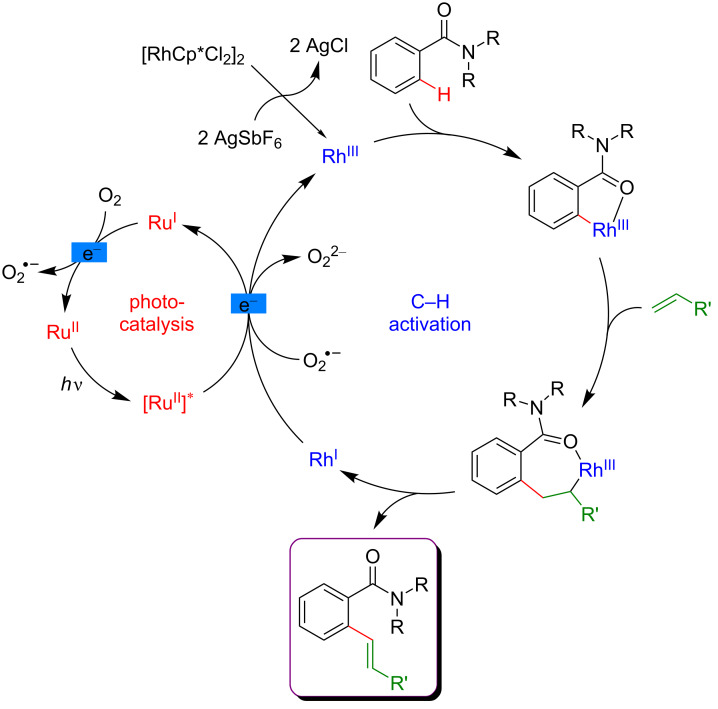
Proposed mechanism for the Heck reaction on arenes via dual catalysis.

This C–H olefination of arenes was performed under aerobic conditions in order to reoxidize the photocatalyst (Figure 8). Interestingly, the desired products were also delivered while using a stoichiometric amount of the photocatalyst in the absence of oxygen, suggesting that a direct electron transfer from the photosensitizer allowed the reoxidation of the active catalyst. However, the participation of molecular oxygen cannot be excluded.

**Figure 9:**
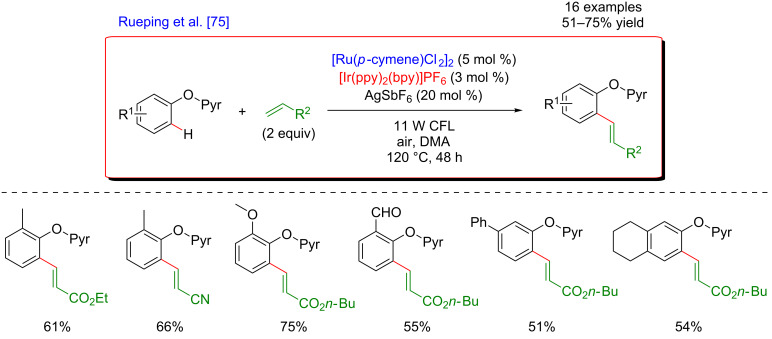
Oxidative Heck reaction on phenols via the dual catalysis.

Rueping further demonstrated the capacity of the dual catalytic systems merging C–H activation with photoredox catalysis to allow in situ reoxidation of the metal catalyst. In 2015, a procedure combining ruthenium C–H activation of phenols derivatives bearing a pyridine moiety as DG and photoredox catalysis was disclosed (Figure 9) [75]. This *ortho*-olefination was performed under air atmosphere and required harsh reaction conditions with temperatures of up to 120 °C. The substituents on both substrates had a minor impact on the reaction outcome and the coupling products were isolated in comparable yields. The applicability of the reaction was underlined by its tolerance towards various functional groups such as aldehydes, ketones, and esters. Of note is that in some cases, partial hydrogenation of the double bond was observed, resulting probably from the generation of a ruthenium–hydride complex. However, this consecutive reactivity and the ratio between the olefin and alkane products could be controlled by adjusting the acidity of the reaction media.

**Figure 10:**
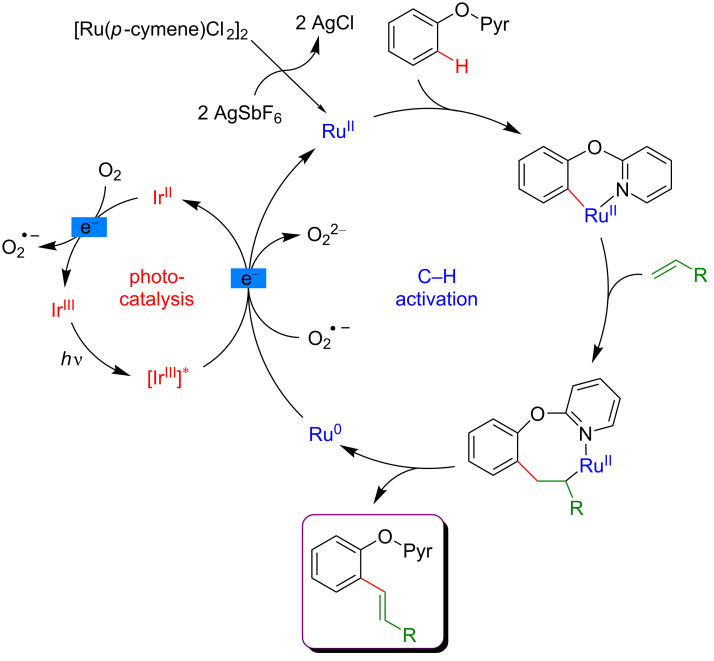
Proposed mechanism for the Heck reaction on phenols via dual catalysis.

As in the previously described examples, molecular oxygen plays a key role in the mechanism of the C–H functionalization (Figure 10). Indeed, both the photoexcited Ir-based catalyst and the superoxide radicals formed in situ from molecular oxygen allowed the reoxidation of the Ru(II) active species for C–H activation.

**Figure 11:**
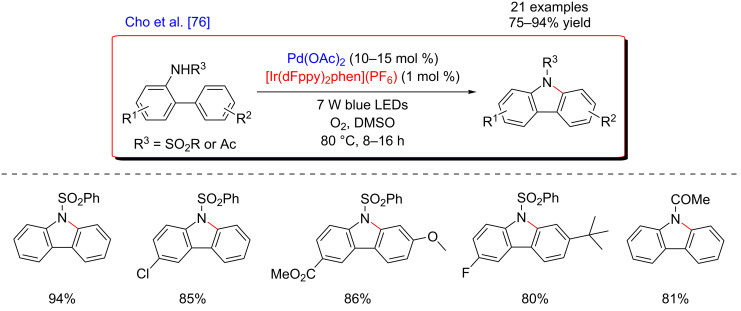
Carbazole synthesis via dual C–H activation/photoredox catalysis.

In 2015, Cho’s group became interested in implementing such mild and sustainable reoxidation protocol in a Pd-catalyzed C–H activation reaction, focusing mainly on an intramolecular direct C–N coupling. Thus, an efficient method for the synthesis of carbazoles via an intramolecular C–H amination was developed, using *N*-substituted 2-aminobiaryl derivatives as the substrates (Figure 11) [76]. This procedure was carried out at 80 °C and led to the formation of various interesting coupling products with excellent yields. Remarkably, a high functional group tolerance was observed, including heterocyclic substrates. In contrast, the *N*-substitution pattern clearly impacted the efficiency of the reaction. Indeed, exchanging the *N*-sulfonyl or *N*-acetyl protecting group with other functionalities, such as hydrogen, benzyl, or alkyl, resulted in the shut of reactivity and no formation of the corresponding *N*-substituted carbazoles was observed.

**Figure 12:**
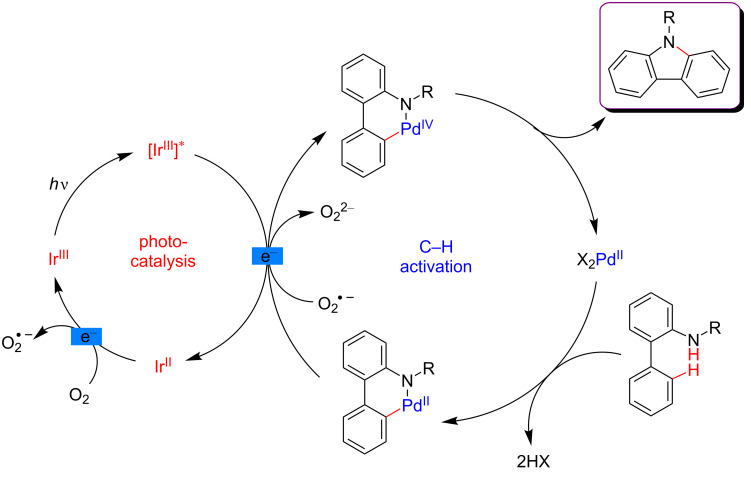
Proposed mechanism for the carbazole synthesis via dual catalysis.

Concerning the mechanism, the carbazole-forming reaction also required aerobic conditions in order to reoxidize the Ir-photocatalyst with molecular oxygen (Figure 12). A similar catalytic cycle than the one reported by Rueping was hence proposed for the C–H activation step. Nevertheless, the authors surmised that an alternative pathway via a Pd(III)/Pd(I) catalytic system could also be envisioned. In this scenario, the reductive elimination from a Pd(III) intermediate occurs prior to the one-electron oxidation by the photoexcited catalyst.

**Figure 13:**
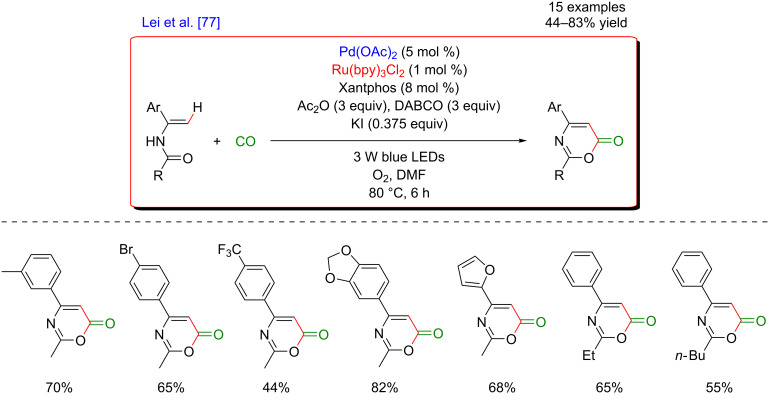
Carbonylation of enamides via the dual C–H activation/photoredox catalysis.

This contribution inspired also other research groups to investigate synergistic catalyses using Pd-based C–H catalysts. In 2016, Lei’s group reported the aerobic intramolecular oxidative carbonylation of enamides for the synthesis of 1,3-oxazin-6-ones by merging palladium and photoredox catalysis (Figure 13) [77]. The transformation was performed at 80 °C under air atmosphere. This efficient approach for the construction of heterocycles exhibited a good functional group tolerance thus providing a broad range of products, isolated in moderate to good yields. A possible gram scale-up synthesis was also accomplished.

**Figure 14:**
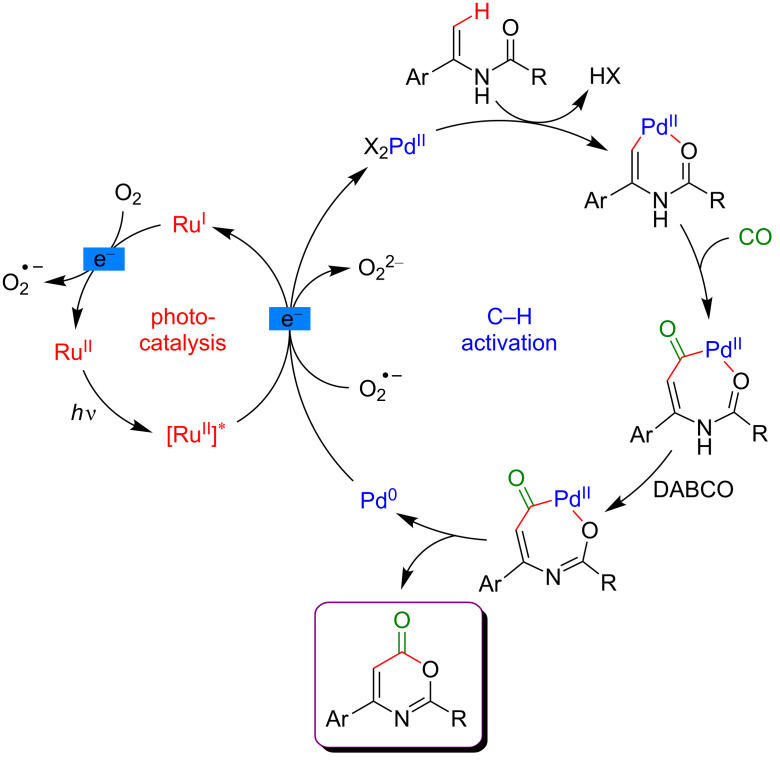
Proposed mechanism for carbonylation of enamides via dual catalysis.

Within the mechanistic cycle, after initial vinylic C–H bond activation and insertion of CO into the C–Pd bond, the acylpalladium intermediate is converted into its conjugated analog under DABCO assistance (Figure 14). The Pd(0) species, liberated during the reductive elimination, is reoxidized via electron transfer with O_2_ playing the role of the terminal oxidant.

**Figure 15:**
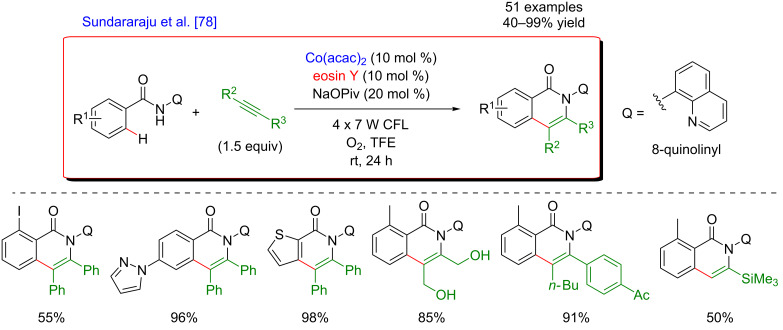
Annulation of benzamides via the dual C–H activation/photoredox catalysis.

In 2018, Sundararaju et al. reported the first example of dual catalysis employing cobalt, a non-noble metal, in combination with a photocatalyst. The strategy was utilized for the C–H bond annulation of benzamides with alkynes, furnishing isoquinolones, an important moiety in various natural products (Figure 15) [78]. The C–H activation step was assisted by an 8-quinolyl DG and occurred under an oxygen atmosphere at room temperature. A variety of benzamides bearing different substituents at the *ortho-* and *para-*positions gave the products in good to excellent yields, while the *meta*-substituted substrates were converted into a mixture of two isomeric products. Moreover, amides containing heterocycles such as thienyl, pyrazolyl, pyridyl, pyrimidyl, and 7-azaindolyl were found to be compatible with this transformation. While symmetrical alkynes led to the desired products in moderate to good yields, the unsymmetrical ones afforded a mixture of products with very high regioselectivity, with the favored formation of cyclized compounds bearing an aromatic unit in α-position to the nitrogen atom. Sensitive functional groups such as hydroxy, silyl, and ethynyl were tolerated by this protocol. Furthermore, this methodology was also extended towards the C–H annulation with dienes.

**Figure 16:**
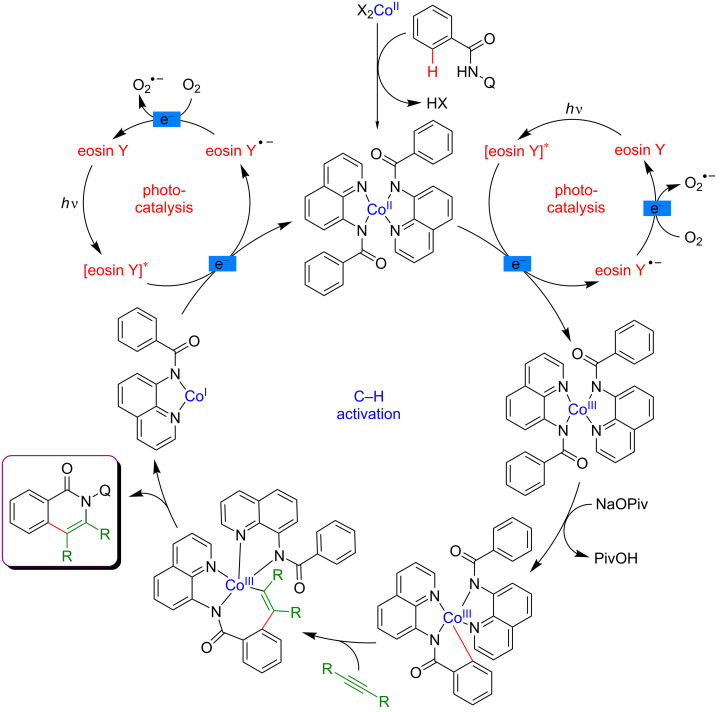
Proposed mechanism for the annulation of benzamides via dual catalysis.

The proposed mechanism of this reaction requiring only oxygen as sole oxidant and implementing two photoredox cycles is presented in Figure 16. After a ligand exchange, Co(II) undergoes a single-electron transfer (SET) oxidation to Co(III) with a concomitant formation of the reduced organic photocatalyst. The thus generated highly reactive Co(III) complex undergoes cyclometalation via a concerted metalation–deprotonation (CMD) process furnishing a cyclometalated intermediate. Thereafter, coordination and subsequent insertion of the alkyne into the Co–C bond delivers a seven-membered cobaltacycle. The desired coupling product is then liberated by reductive elimination, producing a Co(I) species. Finally, the photoexcited eosin Y reoxidizes Co(I) to Co(II), while the photosensitizer is reoxidized by molecular oxygen, thus completing the overall catalytic process.

**Figure 17:**
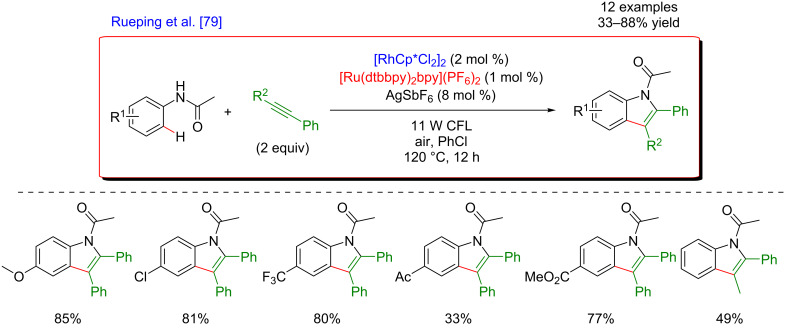
Synthesis of indoles via the dual C–H activation/photoredox catalysis.

In addition, a complementary strategy towards photocatalytic C–H annulation was disclosed by Rueping et al., allowing the synthesis of *N*-heterocycles such as indoles and pyrroles. The targeted heterocyclic products were obtained by cyclization of acetanilides with alkyne derivatives via a direct *ortho*-metalation pathway (Figure 17) [79]. Acetyl substituents acted as DG in this transformation performed under air at high temperatures. The coupling exhibited a broad substrate scope and the procedure tolerated a variety of even sensitive functional groups, including trifluoromethyl, ketone, or ester motifs. Unsymmetrical alkynes were also compatible, although the cyclization provided the desired heterocyclic compounds in decreased yields.

**Figure 18:**
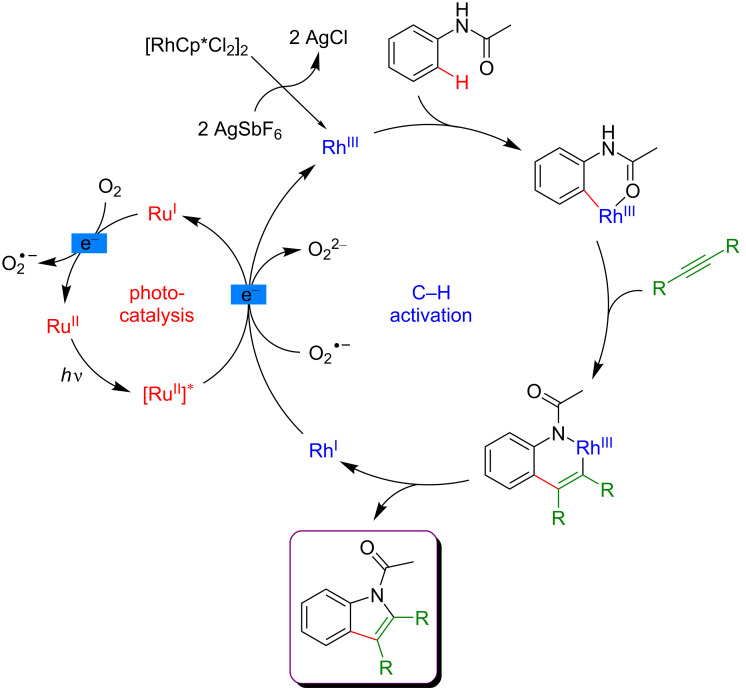
Proposed mechanism for the indole synthesis via dual catalysis.

Once again, the use of Ru-based photocatalyst in combination with molecular oxygen as a terminal oxidant was crucial in this cyclization/C–H functionalization pathway (Figure 18). The classical C–H activation cycle involves an initial amide-directed *ortho-*metalation, followed by the insertion of an alkyne into the C–M bond. The key step of the dual catalysis is the reductive quenching of the photoexcited catalyst by Rh(I), a low-valent metal obtained after C–N reductive elimination. Hence, the Rh(III) active catalyst is regenerated and the resulting reduced photocatalyst is reoxidized to its ground state by molecular oxygen, thus closing both catalytic cycles. Remarkably, this transformation could also be efficiently performed with heterogeneous inorganic photocatalysts, as was illustrated by a reaction performed in the presence of WO_3_.

The above-presented transformations are appealing as the use of stoichiometric amounts of a metal-based oxidant could be obviated with these dual catalysis strategies. Furthermore, in terms of the reactivity, the previously mentioned examples can be rather considered as improved, more sustainable versions of the known C–H methodologies.

### Synergistic catalysis (activation of each coupling partner by one type of catalysis)

**Figure 19:**
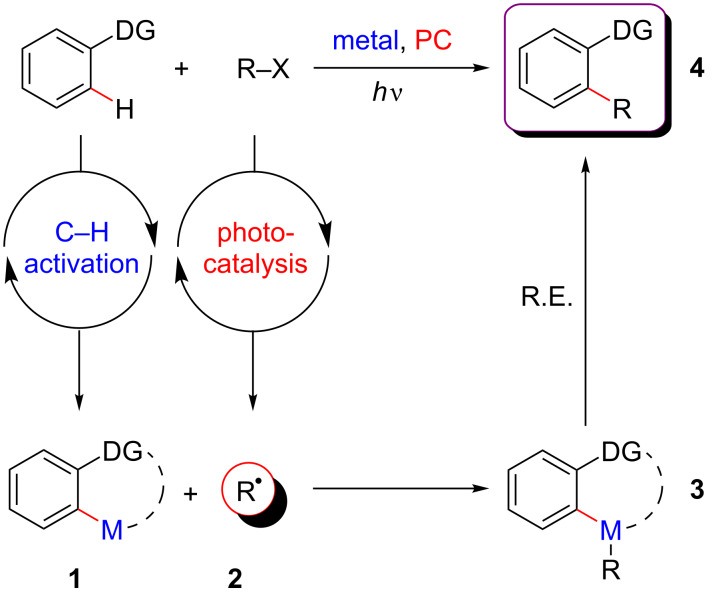
General concept of dual catalysis merging C–H activation and photoredox catalysis.

Synergistic catalysis refers to a dual catalytic system merging C–H bonds activation and visible-light photocatalysis. Following the definition of the synergistic catalysis presented in the introduction, both catalytic cycles enable the distinct activation of each coupling partner in a cooperative fashion (Figure 19). Such transformations will thus typically consist in the insertion of a transition metal into a C–H bond to produce a metalacyclic intermediate **1** which can intercept a radical **2** generated by photoredox catalysis. This interception leads to a new metallic key intermediate **3** by single-electron transfer (SET). The desired coupling product **4** is then obtained after a reductive elimination (Figure 19). Applying such an approach paved the way towards unprecedented couplings benefiting from a SET activation of one substrate, and a two-electron activation by a transition-metal catalyst of the other.

The main advantage of such a system is to promote highly regioselective reactions. Indeed, while radical transformations generally suffer from selectivity issues, interception of radicals by metal catalysts and further cross-coupling-type reactivity provides a unique tool to solve this critical issue. Besides, dual catalytic systems enable also umpolung-type reactivity, thus promoting unique bond-forming reactions, inaccessible via standard approaches due to the polarity mismatch. Finally, the combination of photocatalysis and metal-catalyzed C–H activations upholds the desired couplings under, usually, mild conditions and at room temperature, while utilization of non-prefunctionalized coupling partners reduces the waste formation. Last but not least, the use of visible light, one of the most economic and abundant source of energy to promote these transformations is in agreement with sustainable and eco-friendly chemistry.

### Directed C–H activation

#### Pd-catalyzed arylation

**Figure 20:**
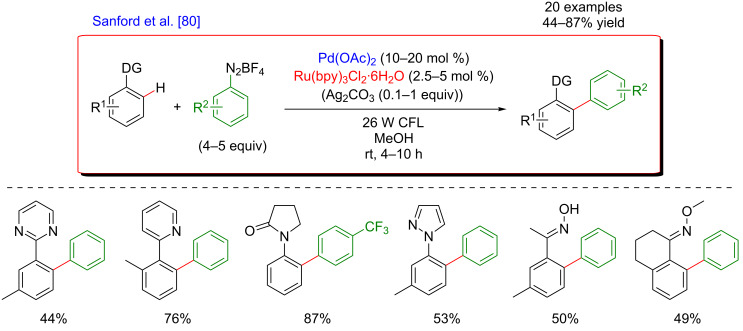
The first example of dual catalysis merging C–H activation and photoredox catalysis.

The first example of dual catalysis merging C–H activation and photoredox catalysis was published in 2011 by Sanford [80]. The transformation concerned the direct arylation of various aromatic compounds bearing different DGs with aryldiazonium salts (Figure 20). In her initial study in 2005, Sanford reported a similar arylation and diazonium salts were already identified as appropriate coupling partners. However, harsh reaction conditions with high temperatures of 100 °C in acetic acid were necessary to promote the reaction [81]. The major enhancement was discovered while surmising that an identical product might be obtained, if the Ar^•^ was generated in situ and used as a highly reactive coupling partner. Such methodology using a dual catalytic system thus provided a significant improvement compared to the initial protocols. Indeed, the reaction was carried out at room temperature, demonstrating the capacity of photoredox catalysis to generate reactive species under exceptionally mild conditions. The combination between the photocatalyst Ru(bpy)_3_Cl_2_·6H_2_O and Pd(OAc)_2_ promoted the formation of the coupling products in high yields when the reaction was irradiated with visible light. This new procedure exhibited a good functional group tolerance and turned out to be compatible with a wide range of DGs, including amides, pyrazoles, pyrimidines, and oximes (Figure 20). The mild arylation proceeded in good to excellent yields over 20 examples and worked with electron-deficient, electron-rich as well as relatively sterically hindered arylating reagents.

**Figure 21:**
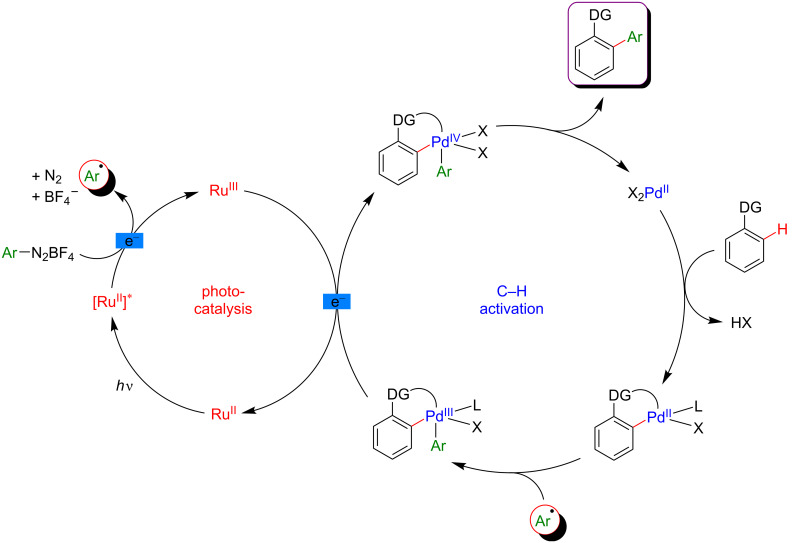
Proposed mechanism for the C–H arylation with diazonium salts via dual catalysis.

The mechanism proposed by the authors is presented in Figure 21. First, the DG-assisted C–H activation of the aromatic compounds by Pd(II) leads to the formation of the corresponding palladacycle. In parallel, the photocatalyst excited by visible light, Ru(II)* reduces the aryldiazonium salt, generating the corresponding aryl radical and the oxidized Ru(III). Then, the highly reactive radical species formed is intercepted by the palladacycle to produce a Pd(III) metallacycle intermediate bearing both coupling partners. This oxidative addition is the key step of the mechanism, thus making this transformation possible. A SET between this metallacycle and the Ru(III), rendering the reaction feasible at room temperature, regenerates the ground-state Ru(II) photocatalyst concomitantly with the formation of a Pd(IV) species. At this point, a reductive elimination provides the desired coupling product together with the Pd(II) catalyst, thus completing this synergistic catalytic system (Figure 21). However, a subsequent mechanistic study indicated that the order of the two last steps might be inversed, thus leading to the reductive elimination on the Pd(III) intermediate followed by the single-electron oxidation of the Pd(I) resulting species [82].

**Figure 22:**
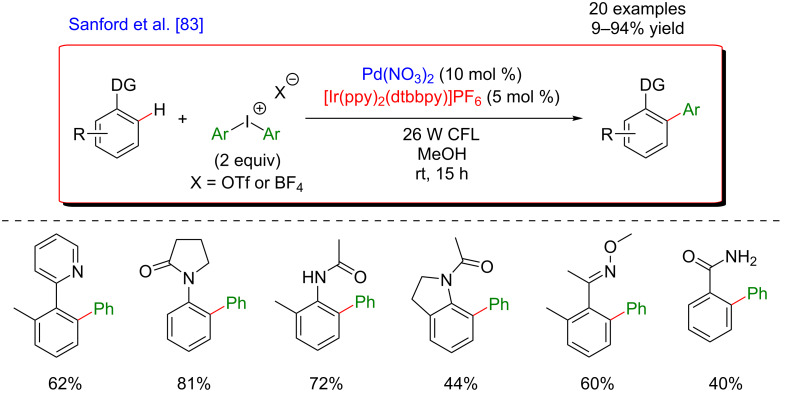
Dual catalysis merging C–H activation/photoredox using diaryliodonium salts.

Thereafter, Sanford’s group showed in 2012 the possibility to extend this C–H activation/photoredox dual catalytic system by using diaryliodonium salts as radical precursors [83]. Considering the higher reduction potentials of these compounds in comparison with the corresponding diazoniums, a stronger reductive iridium-based photocatalyst was necessary to generate the aryl radicals (Figure 22). Similar results were obtained with this new procedure. This modified protocol was also compatible with a diversity of DGs and diazonium salts, thus furnishing a panel of 20 biaryls in good yields.

Inspired by this work, several research groups independently published alternative procedures based on the same dual catalytic system merging C–H activation and photoredox catalysis.

**Figure 23:**
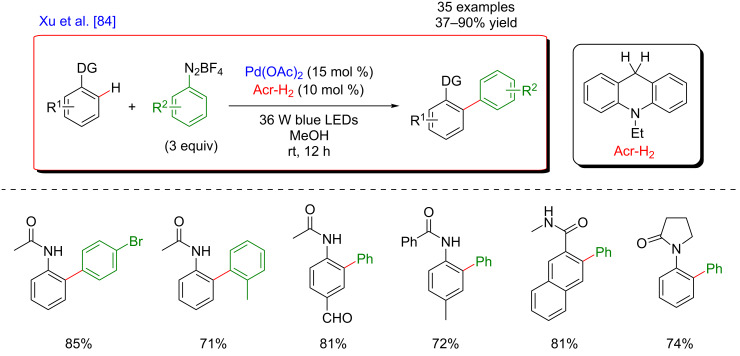
Direct arylation via the dual catalytic system reported by Xu.

In 2017, Xu’s group reported a variation of the *ortho*-arylation of acetanilides and benzamides with aryldiazonium salts [84], now compatible with a cheaper photosensitizer, i.e., the organic Acr–H_2_ molecule (Figure 23). In this study, 35 examples of the targeted coupling products were afforded in good to excellent yields. Various synthetically relevant functional groups, including halides, esters, amides, ethers, ketones, and aldehydes were compatible with the protocol. This outcome is interesting from the sophisticated molecule late-stage functionalization viewpoint. Furthermore, the quantum yield of the reaction was measured and turned out to be 6.5. This result indicated that the mechanism of this reaction involved a short radical chain process [85].

**Figure 24:**
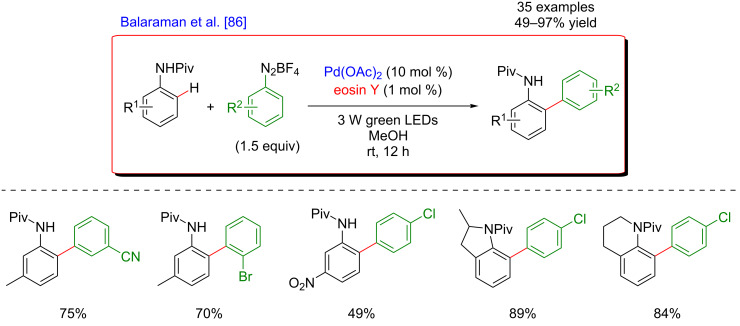
Direct arylation via dual catalytic system reported by Balaraman.

The same year, a similar arylation was disclosed by Balaraman et al. using eosin Y as a photoredox catalyst (Figure 24) [86]. This methodology operated under mild conditions, was efficient using a low loading of both catalysts and could be scaled-up to a gram-scale. The procedure featured a good functional tolerance and was compatible with a wide range of both substrates, thus delivering 35 examples of coupling products in good to excellent yields. Furthermore, the authors easily accomplished the removal of the DG and performed the diversification of *ortho*-arylaniline derivatives products into synthetically relevant *N*-heterocyclic compounds such as phenanthridine, carbazole, and dibenzo[*b,d*]azepine.

**Figure 25:**
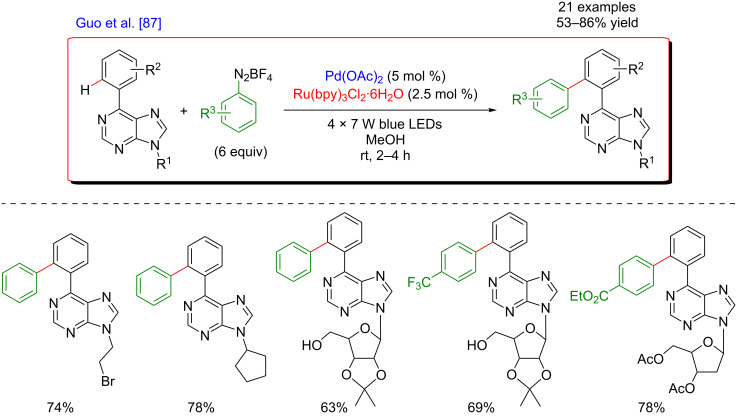
Direct arylation via dual catalytic system reported by Guo.

Simultaneously, Guo’s group published the arylation of nucleosides bearing a purine moiety. The transformation was regioselective and tolerated various substituents at the N9 position of the purine, including sugar units (Figure 25) [87]. Interestingly, in this approach, the purine heterocycle acted as an inherent DG for the C–H activation step. The protocol provided a broad scope of products featuring good functional group tolerance, could be performed on a gram scale and occurred under mild conditions, essential features when working with biological systems. Thus, a variety of functionalized purines nucleosides that are potentially of great importance in medicinal chemistry were obtained in good yields.

**Figure 26:**
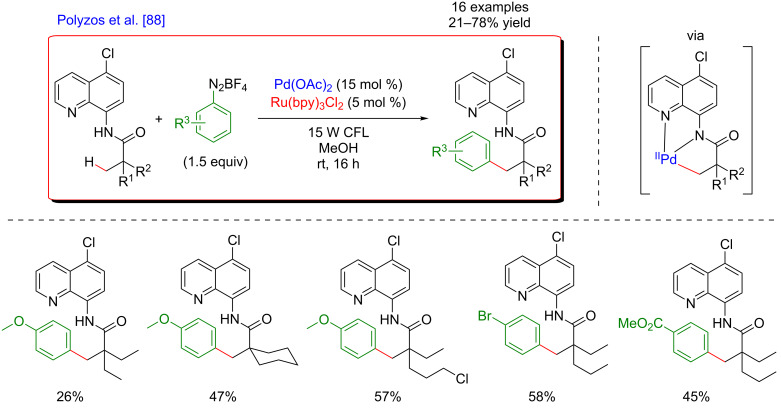
C(sp^3^)–H bond arylation via the dual Pd/photoredox catalytic system.

In contrast to C(sp^2^)–H bonds, C(sp^3^)–H bond activation is much more challenging. Inspired by the potential of bicoordinating DGs to facilitate C(sp^3^)–H metalation, Polyzos’s group reported the direct arylation of aliphatic bonds with aryldiazonium salts via a palladium/photoredox dual catalysis (Figure 26) [88]. The method utilized an 8-aminoquinoline-derived bidentate auxiliary, and, in contrast with a vast majority of C(sp^3^)–H bond activation reactions, was effective at room temperature. The coupling displayed high selectivity for β-methyl C(sp^3^)–H bonds. Of note is that a substitution at the C5-position of the 8-aminoquinoline ring was beneficial for the C–H activation step as the functionalization of 5-chloro-8-aminoquinoline delivered the arylated products in increased yields. Carbocyclic rings, long alkyl chains, methoxy, chloride, and phenyl groups were tolerated on the C–H substrates and electron-poor as well as electron-rich diazonium salts could be coupled smoothly.

#### Pd-catalyzed acylation

**Figure 27:**
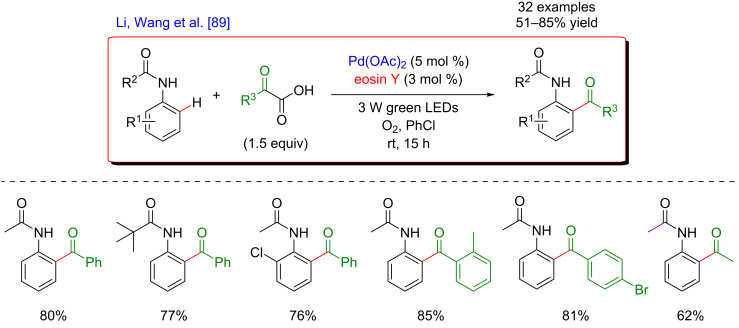
Acetanilide derivatives acylation via the dual C–H activation/photoredox catalysis.

The dual catalytic system merging C–H activation and photoredox catalysis was also applied to C(sp^2^)–H acylation reactions via the generation of acyl radicals. In 2015, Li, Wang and co-workers reported the *ortho*-acylation of acetanilide derivatives using α-ketoacids as coupling partners and employing synergistic catalysis combining palladium-catalyzed C–H activation and photoredox catalysis (Figure 27) [89]. Interestingly, the transformation occurred smoothly in the presence of eosin Y, an inexpensive (in comparison with Ir- or Ru-based photoredox catalysts), and easily accessible organic photocatalyst. The reaction, carried out at room temperature, promoted the introduction of various aromatic as well as aliphatic acyl motifs and was applicable to a broad range of substrates hence furnishing more than 30 acylated acetanilides in good to excellent yields.

**Figure 28:**
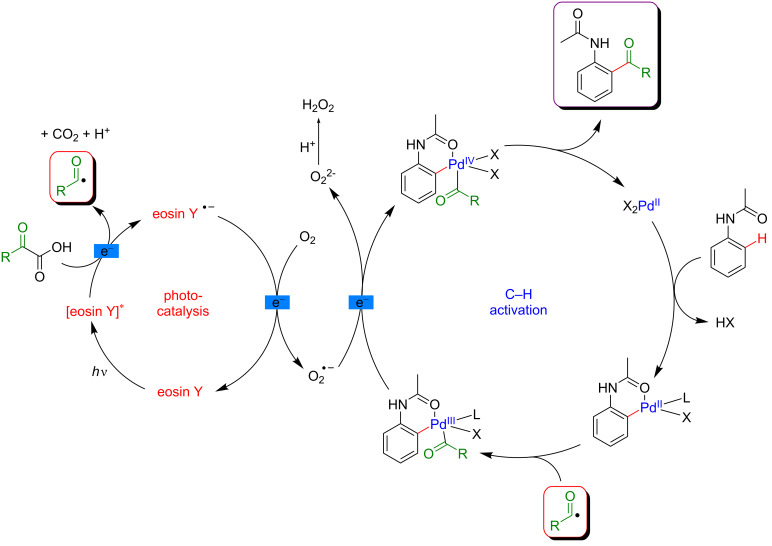
Proposed mechanism for the C–H acylation with α-ketoacids via dual catalysis.

The proposed mechanism involves an initial amide-directed C–H activation step to produce a palladacyclic intermediate. In parallel, the oxidative decarboxylation of the α-ketoacid induced by the photocatalyst in its excited state generates the corresponding acyl radical. The latter is intercepted by the palladacycle, leading to a Pd(III) species. Remarkably, the molecular oxygen enables to close the catalytic cycle by sequentially oxidizing the photoredox catalyst and the Pd(III) species via the formation of superoxide anion. In this way, the photocatalyst is regenerated in its ground state and the Pd(IV) intermediate undergoes fast reductive elimination, liberating the expected product (Figure 28).

**Figure 29:**
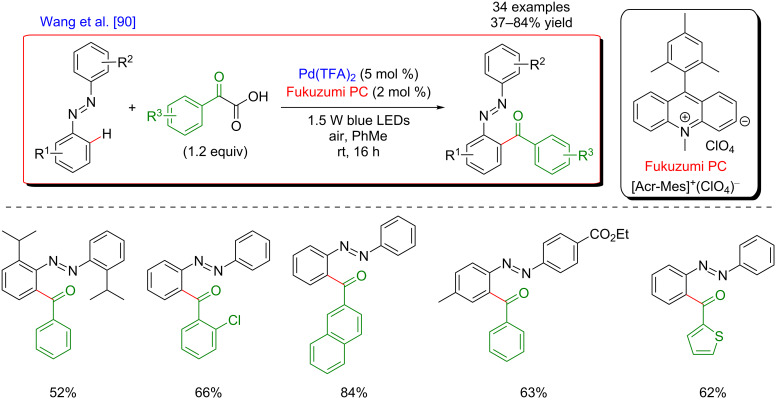
Acylation of azobenzenes via the dual catalysis C–H activation/photoredox.

In 2016, Wang et al. further demonstrated the possibility of extending this approach to the C(sp^2^)–H acylation of azo- and azoxybenzene derivatives (Figure 29) [90]. Similarly, acyl radicals generated by photodecarboxylation of α-ketoacids were engaged in a dual catalytic system. The strategy used the Fukuzumi acridinium salt organic photocatalyst and Pd(TFA)_2_ to promote the C–H activation step. In this case, the transformation was only compatible with aromatic α-ketoacids. Nevertheless, the reaction was performed at room temperature and tolerated a wide spectrum of both substrates bearing various functional groups, thus furnishing more than 30 acylated products in moderate to good yields. As the molecular oxygen was the terminal oxidant, the typical high loading of an external oxidant could be avoided.

Flow chemistry is an important technology for photochemical reactions due to the inherent lack of scalability issues as a consequence of the Beer–Lambert law of absorption. Consequently, continuous-flow chemistry represents one of the few ways in which pharmaceutically relevant quantities of compounds can be synthesized through photoinduced transformations [91]. In this context, great advances were also recently achieved in the field of direct C–H functionalization reactions under flow conditions. Persistent improvements enabled the development of innovative flow techniques, encompassing large-scale photochemical procedures, which were successfully applied to various C–H functionalization reactions [92–93].

**Figure 30:**
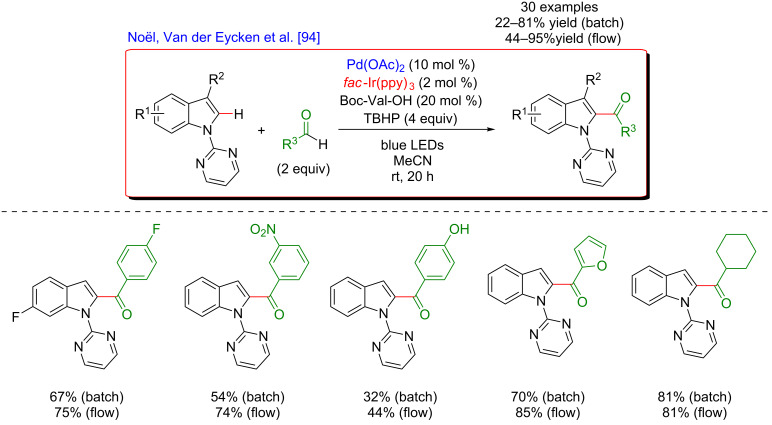
C2-acylation of indoles via the dual C–H activation/photoredox catalysis.

In 2017, Noël, Van der Eycken and co-workers hence astutely combined the dual catalysis strategy with flow microreactor technology to achieve C2-acylation of indole derivatives with aldehydes (Figure 30) [94]. Both electron-rich and electron-poor aromatic or aliphatic aldehydes were potent coupling partners for this procedure. The transformation also allowed the introduction of heterocyclic moieties at the C2-position of indoles, hence exhibiting a wide substrate scope of this coupling.

**Figure 31:**
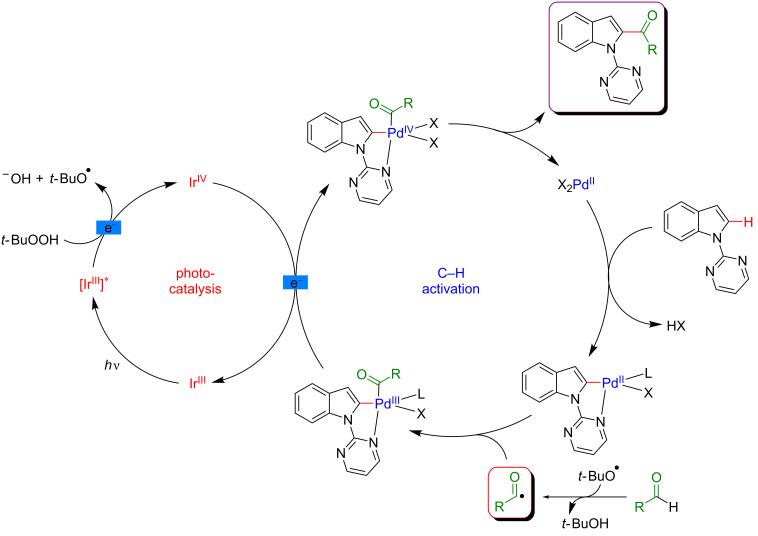
Proposed mechanism for the C2-acylation of indoles with aldehydes via dual catalysis.

The proposed mechanism of this acylation is shown in Figure 31. As previously, distinct catalytic cycles involving photoredox catalysis and C–H activation act in a cooperative way to promote the formation of a new bond. The strategy is based on the presence of a pyrimidine DG at the N1-position of the indole in order to facilitate the C–H activation step. Furthermore, a peroxide (TBHP) is needed to oxidize the Ir-based photoredox catalyst and to generate the acyl radical via hydrogen atom transfer. From the mechanistic perspective this synergistic dual catalytic system merging C–H activation and photocatalysis is similar to the one described by Sanford for the arylation with diazonium salts.

**Figure 32:**
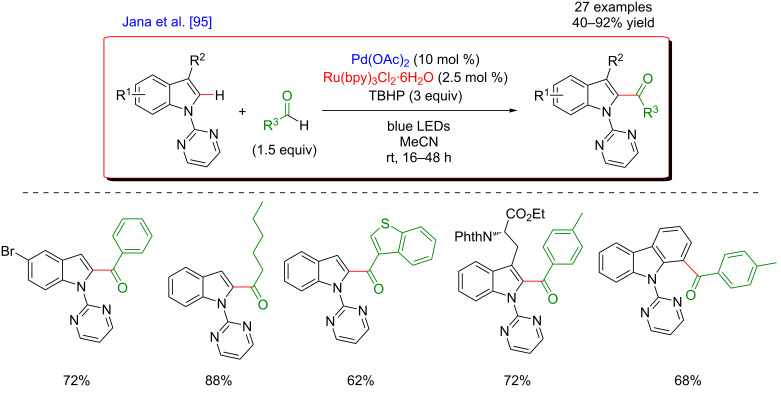
C2-acylation of indoles via the dual C–H activation/photoredox catalysis.

The same year, Jana’s group independently reported an analogous approach allowing the C2-acylation of indole derivatives with aldehydes (Figure 32). The only difference in comparison with Noël and Van der Eycken’s procedure concerned the nature of the photocatalyst used for the transformation, which was a Ru-based photosensitizer in that case [95]. This time, the acylation reaction was exclusively performed in batch and the protocol was compatible with various substrates bearing synthetically useful functional groups. Numerous coupling products were hence obtained in good yields and the authors applied the methodology for the late-stage acylation of natural ʟ-tryptophan as well as carbazole derivatives.

#### Cu-catalyzed transformations

Sporadic examples using copper as transition metal for C–H functionalization reactions in combination with photocatalysis were also reported in the literature. The key advantages of the use of Cu as catalyst are related to its great abundance, as well as its cheap and non-toxic character [14,96–97].

**Figure 33:**
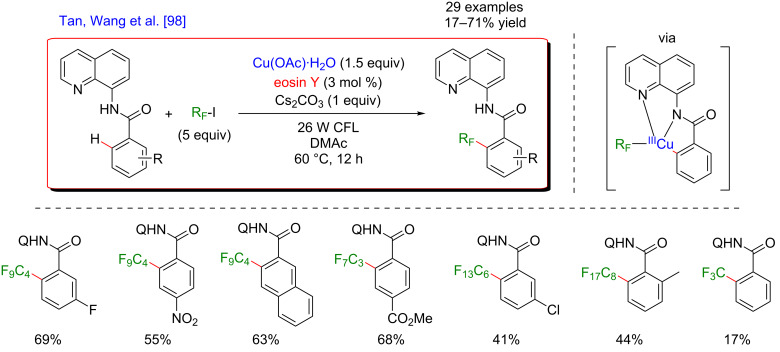
Perfluoroalkylation of arenes via the dual C–H activation/photoredox catalysis.

In 2016, Tan, Wang and co-workers developed a methodology allowing the *ortho*-perfluoroalkylation of benzamides. This reaction photoinduced by visible light was promoted by copper and used the bidentate 8-aminoquinoline moiety as the DG (Figure 33) [98]. Remarkably, eosin Y, a cheap organic photocatalyst, enhanced the generation of perfluoroalkyl radicals from the corresponding alkyl iodides but a stoichiometric amount of a simple copper salt, acting as both, the catalyst for C–H activation and the oxidizing agent, was also required. The reaction occurred at higher temperatures (60 °C) compared to other dual catalytic systems. A wide variety of benzamide substrates bearing different functional groups such as alkyl, phenyl, halogen, ethoxy, trifluoromethyl, nitro, and ester underwent efficiently the desired perfluoroalkylation. This coupling was sensitive to steric hindrance as *ortho-*substituted substrates were less efficiently converted into the desired products. Furthermore, various perfluoroalkyl iodides with different chain lengths were potent substrates, affording the corresponding perfluoralkylated products in moderate to good yields, except for trifluoromethyl iodide, presumably due to the low boiling point of CF_3_I. Finally, the removal of the DG allowed the post-modification of the products into the corresponding perfluoroalkyl benzoic acid derivatives.

**Figure 34:**
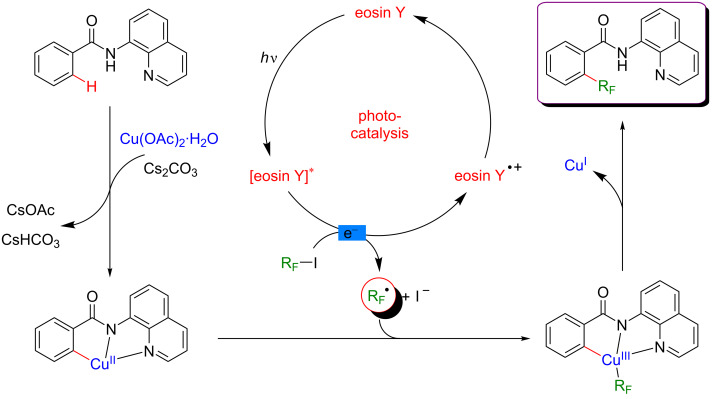
Proposed mechanism for perfluoroalkylation of arenes via dual catalysis.

A plausible mechanism for the perfluoroalkylation of *N*-(quinolin-8-yl)benzamides is proposed in Figure 34. First, C–H activation of the benzamide substrate with copper produces a cyclometalated complex. Meanwhile, perfluoroalkyl radicals are generated by electron transfer between the perfluoroalkyl iodides and the excited photoredox catalyst. Interception of the perfluoroalkyl radical by the Cu(II) complex delivers a new Cu(III) metalacyclic intermediate which releases the desired *ortho*-functionalized product via reductive elimination.

**Figure 35:**
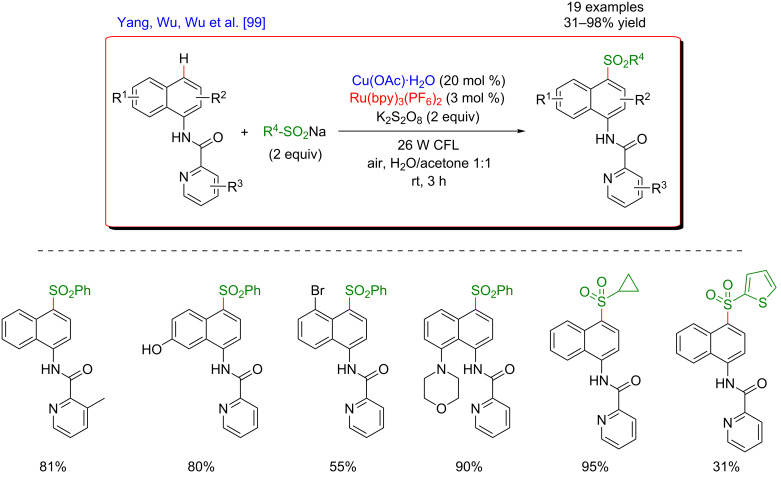
Sulfonylation of 1-naphthylamides via the dual C–H activation/photoredox catalysis.

In 2017, Yang, Wu, Wu et al. reported a mild and efficient protocol for the remote C4–H sulfonylation of 1-naphthylamine derivatives with sodium sulfinates in the presence of K_2_S_2_O_8_ as the oxidant (Figure 35) [99]. This strategy was based on the use of a bidentate picolinamide DG promoting the direct C–H functionalization of the naphthalene ring. The transformation proceeded under either Cu/Ag co-catalysis or using the dual Ru/Cu photoredox system. In the latter case, the methodology was operative at room temperature within a short reaction time, affording the desired products in good to excellent yields. The described sulfonylation procedure was general and both electron-withdrawing or -donating substituents were well tolerated at different positions of the 1-naphthylamine derivatives. Aromatic, aliphatic as well as heterocyclic sodium sulfinates were also suitable coupling partners for this C–H functionalization. In addition, the reaction occurred smoothly on a gram scale and the DG could be subsequently removed by hydrolysis, further demonstrating the synthetic potential of this protocol.

**Figure 36:**
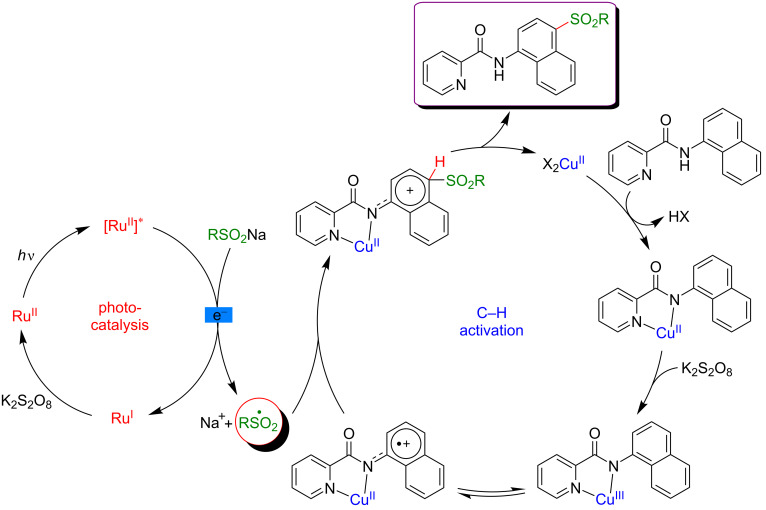
Proposed mechanism for sulfonylation of 1-naphthylamides via dual catalysis.

The authors proposed a plausible mechanism involving a radical pathway and suggested that this C4–H sulfonylation reaction might proceed via a single electron-transfer process (Figure 36). In the first place, a bidentate chelated species is formed by the coordination of 1-naphthylamine derivatives with copper salt. The subsequent oxidation of this intermediate with potassium persulfate produces a Cu(III) species, furnishing a radical cation on the naphthyl ring after an intramolecular electron transfer. In the meantime, the sulfonyl radical is generated by oxidation of the sulfinate substrate via the photocatalytic cycle with the Ru(bpy)_3_ catalyst and K_2_S_2_O_8_ (the direct oxidation of sulfinate by persulfate could also be envisioned). Thereafter, a cationic intermediate is afforded by the radical combination between the sulfonyl radical and the 1-naphthyl derivative at the C4 position. Lastly, a proton-transfer process and dissociation result in the formation of the expected coupling product, accompanied by the regeneration of the Cu salt to close the catalytic cycle.

#### Direct metalation and visible-light absorption by a single catalyst: Ru and Rh-catalyzed photoinduced transformations

The dual catalytic systems, combining visible-light photocatalysis and C–H activation generally require the use of two distinct metal catalysts, one able to activate a poorly reactive C–H bond and the second one harvesting the light in a photoredox process. Therefore, the design of alternative approaches, in which a single catalyst can play a double role, i.e., absorbing light and promoting bond-breaking/bond-forming events, seems extremely appealing as such metallaphotocatalysis could thus be envisioned under exogenous photosensitizer-free conditions.

The pioneering work in this extremely challenging research field was reported concomitantly by the groups of Ackermann [100] and Greaney [101], and concerned unique Ru-catalyzed *meta-*selective C–H alkylation of arenes. The ruthenium-catalyzed *meta-*selective C–H functionalization through arene σ-activation was already well established yet limited by harsh reaction conditions and elevated reaction temperatures. Both research groups hence hypothesized that the Ru-metallacyclic intermediate, generated via directed *ortho*-metalation should exhibit visible-light absorption property and thus, under visible-light irradiation, the overall catalytic cycle could benefit from the photocatalytic activation.

**Figure 37:**
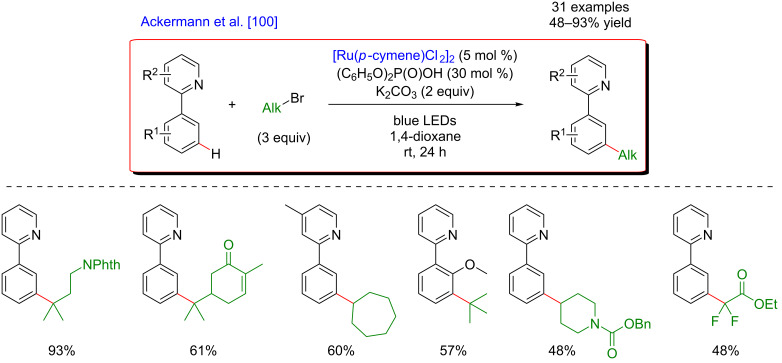
*meta*-C–H Alkylation of arenes via visible-light metallaphotocatalysis.

**Figure 38:**
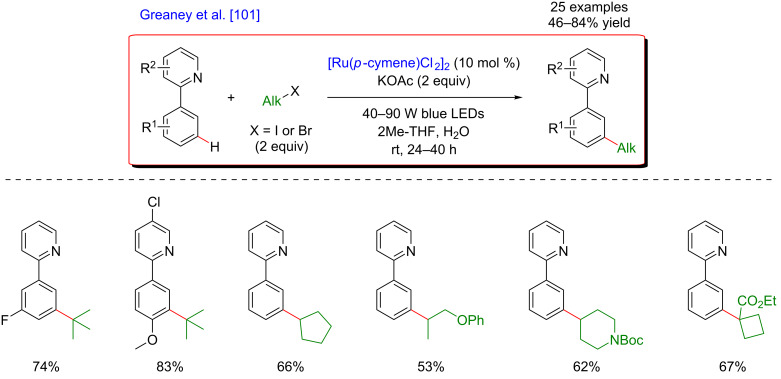
Alternative procedure for *meta*-C–H alkylation of arenes via metallaphotocatalysis.

Following the initial hypothesis, the direct functionalization of phenylpyridine substrates turned out to be very efficient at room temperature while applying blue LED irradiation. Two slightly modified catalytic systems composed of a [Ru(*p*-cymene)Cl_2_]_2_ catalyst and either K_2_CO_3_ or KOAc bases promoted the direct *meta-*selective C(sp^2^)–H alkylation with secondary and tertiary alkyl halides, delivering the expected products in good to excellent yields (Figure 37 and Figure 38). The addition of diphenylphosphoric acid as a ligand guaranteed higher yields and a broader reaction scope [100], including C–H activation substrates bearing alternative and modifiable DGs such as pyrazoles or oxazolines, as well as synthetically meaningful σ-bromo esters. The mildness of the reaction conditions warranted tolerance of sensitive and/or biologically relevant sugar, menthol, and steroid motifs (Figure 37).

**Figure 39:**
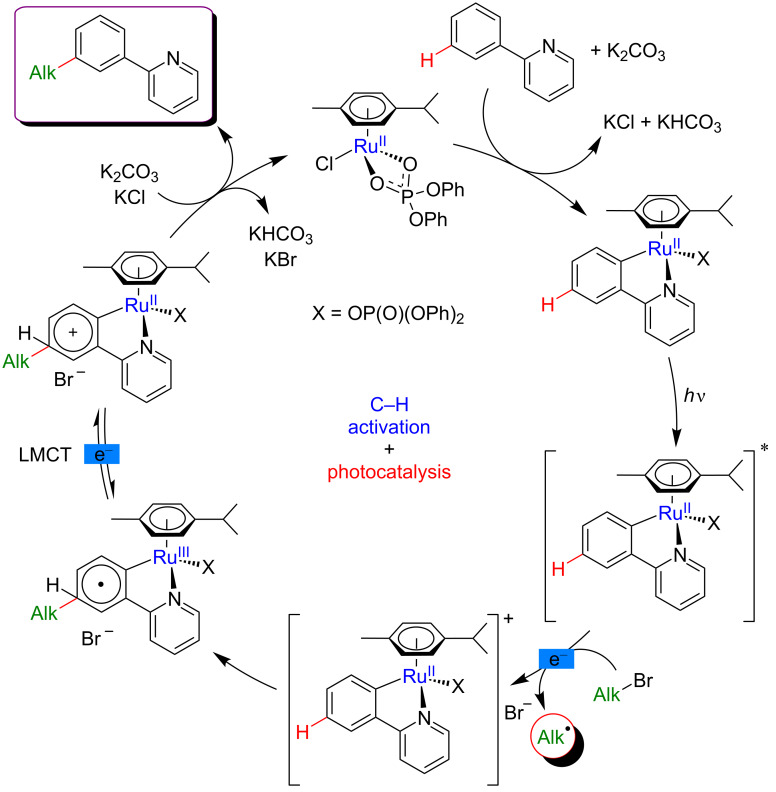
Proposed mechanism for *meta*-C–H alkylation of arenes via metallaphotocatalysis.

The mechanistic studies conducted by Ackermann et al. clearly showed that the metallacyclic intermediates resulting from the C–H cleavage step slightly, yet significantly absorbed light in the relevant blue region. This absorption was believed to trigger the overall catalytic cycle at room temperature as no product formation was observed in the dark. The mechanistic scenario thus foresees an initial pyridine-directed, *ortho*-C–H activation delivering the photoactive intermediate. Under visible-light irradiation, the SET process from the excited ruthenacycle to the haloalkane coupling partner leads to the formation of a stabilized alkyl radical (Figure 39). Next, radical attack at the *para*-position of the carbohydrate ring of the phenylpyridine substrate takes place. Final SET and subsequent rearomatization complete the catalytic cycle, delivering the expected *meta*-functionalized product and regenerating the catalytically competent ruthenium(II) species.

**Figure 40:**
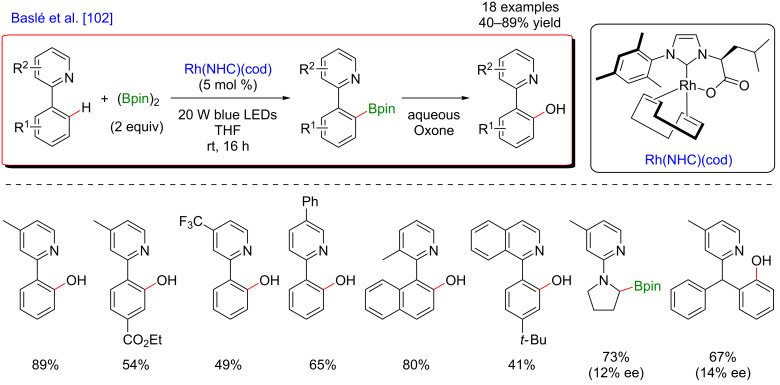
C–H borylation of arenes via visible-light metallaphotocatalysis.

A few months later, Baslé et al. exploited the same concept of merging C–H activation and photocatalysis while using a single metal catalyst. In this case, an original Rh–NHC complex was used for the *ortho-*directed C–H borylation of phenylpyridine substrates (Figure 40) [102]. As previously, the irradiation with visible light allowed performing this challenging borylation under mild reaction conditions, thus delivering, after oxidation of the crude reaction mixture with Oxone, a large panel of the corresponding hydroxylated pyridine products.

**Figure 41:**
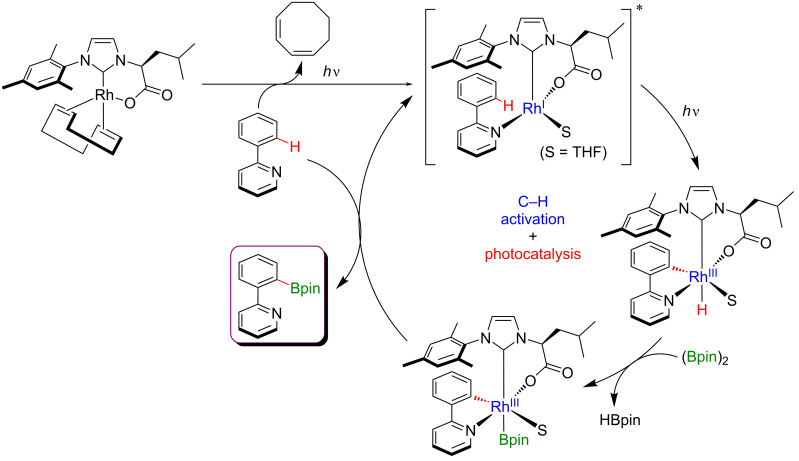
Proposed mechanism for C–H borylation of arenes via visible-light metallaphotocatalysis.

From the mechanistic point of view, this reaction is expected to differ from the previously described Ru-catalyzed *meta*-selective C–H alkylations by the nature of the metal-based photoactive species (Figure 41). Indeed, the NHC–Rh complex is believed to be photoactive after coordination of the phenylpyridine substrate. The generated coordination complex becomes excited after the absorption of visible light and thus undergoes an oxidative *ortho*-C–H addition step via a metal-to-ligand charge transfer process, delivering the Rh(III) hydride species. Following borylation of the Rh(III) intermediate and subsequent reductive elimination liberate the expected coupling product with concomitant regeneration of the catalytically active NHC–Rh(I) complex.

The potential of the above-mentioned reactions is particularly interesting as indeed, a unique, double reactivity might be attributed to a single precatalyst with the in situ formation of photoactive intermediates. Remarkably, the generated metallaphotoredox catalyst is able to harvest visible light and triggers the expected transformation at room temperature.

### Undirected C–H activation

In parallel to the development of the field of metal-catalyzed directed C–H activation, reactions involving the direct functionalization of simple aromatic compounds, depleted of a coordinating motif, have also attracted significant scientific interest [103]. Such couplings allowed constructing molecular complexity from very simple arenes such as benzene, toluene or mesitylene. However, such transformations frequently required rather harsh reaction conditions as well as the addition of strong oxidants and/or additives.

**Figure 42:**
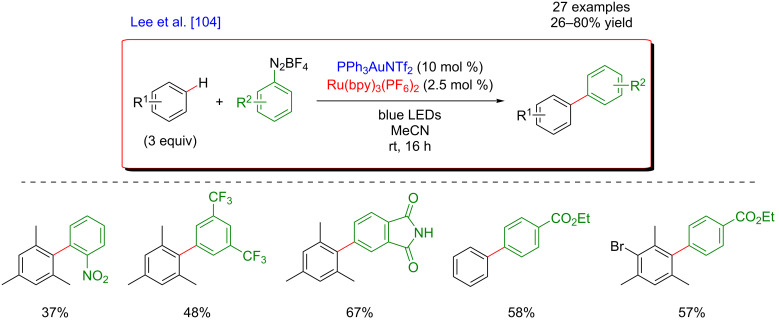
Undirected C–H aryl–aryl cross coupling via dual gold/photoredox catalysis.

In order to provide a solution towards these limitations of the undirected C–H activation, the group of Lee initiated a project merging gold-catalyzed C–H activation with photoredox catalysis (Figure 42) [104]. Such a dual catalytic system should promote the desired C–C bond-forming reaction under oxidant-free conditions, while the use of the gold catalyst gives promise of accessing highly site-selective transformations. Indeed, electrophilic Au(III) species were able to site-selectively activate C–H bonds of activated electron-rich arenes thus generating Au(III)–Ar species.

Following this hypothesis, the desired undirected C–H arylation occurred smoothly between mesitylene (and several other electron-rich aromatic substrates) and aryldiazonium salts as coupling partner, using an Au(I) catalyst in combination with Ru(bpy)_3_(PF_6_)_2_ as the photosensitizer under blue LED irradiation. Remarkably, the test reactions clearly indicated the low efficiency of this Ar–Ar cross-coupling in the absence of the photosensitizer and/or in the dark, while an unselective transformation took place when removing the Au catalyst. Under the optimized reaction conditions, this dual catalysis protocol was compatible with a panel of electron-poor aryldiazonium partners, affording the targeted biaryls in moderate to high yields.

**Figure 43:**
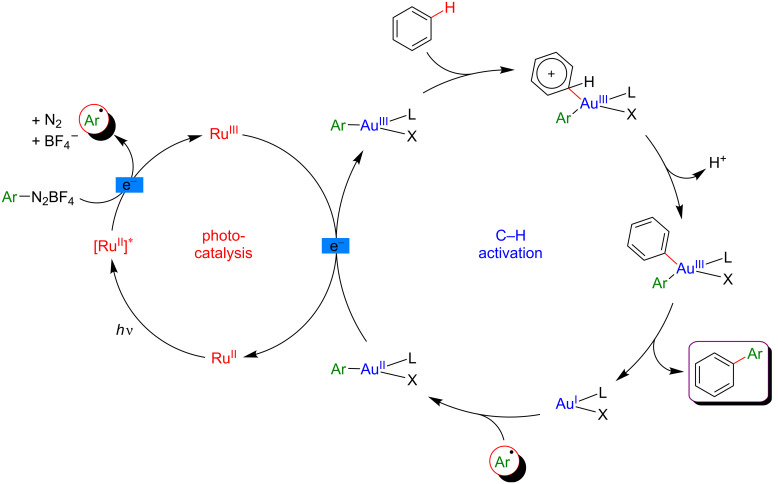
Proposed mechanism for the undirected C–H aryl–aryl cross-coupling via dual catalysis.

Based on a literature survey, a plausible mechanism of this reaction is initiated by the addition of an aryl radical (generated from the corresponding diazonium precursor) to the Au(I) catalyst, followed by SET oxidation to provide an Ar–Au(III) intermediate (Figure 43). This Lewis-acidic Au species promotes the regioselective C–H auration of the electron-rich substrate, delivering a cationic intermediate that under deprotonation and subsequent reductive elimination furnishes the expected biaryl product. In parallel, the photocatalytic cycle warrants the mild generation of the Ar^•^ radical as well as the key Au(II)/Au(III) oxidation.

**Figure 44:**
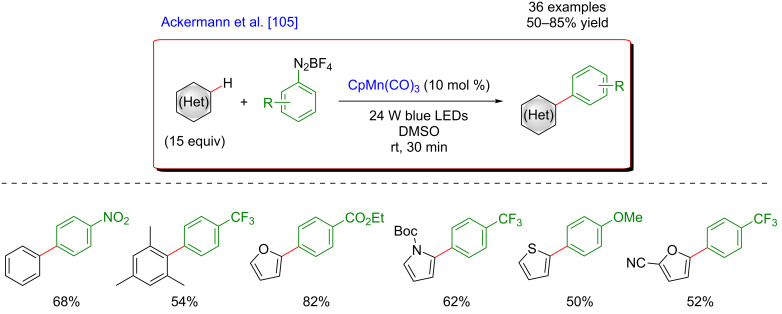
Undirected C–H arylation of (hetero)arenes via dual manganese/photoredox catalysis.

More recently, an alternative strategy for the straight, non-directed arylation of (hetero)arenes was reported by Ackermann (Figure 44) [105]. Targeting more sustainable protocols, a manganese catalyst was selected to trigger the coupling between an aryldiazonium salt and simple (hetero)aryls. A unique reactivity was obtained under irradiation with blue LEDs and a continuous-flow process allowed achieving higher reactivity. Under the optimized reaction protocol, a range of biaryls was synthesized in moderate to good yields under very mild reaction conditions. Nevertheless, due to regioselectivity issues, this photo-flow direct arylation was mainly limited to symmetrical aryls. The site-selectivity issues could, however, be solved by using heteroarene coupling partners. Indeed, furans, thiophenes, and pyrroles underwent selectively the non-directed C–H functionalization, delivering a diversity of heteroaryl–aryl coupling products. Remarkably, the continuous-flow technique rendered this transformation perfectly suited for large-scale and rapid synthesis.

**Figure 45:**
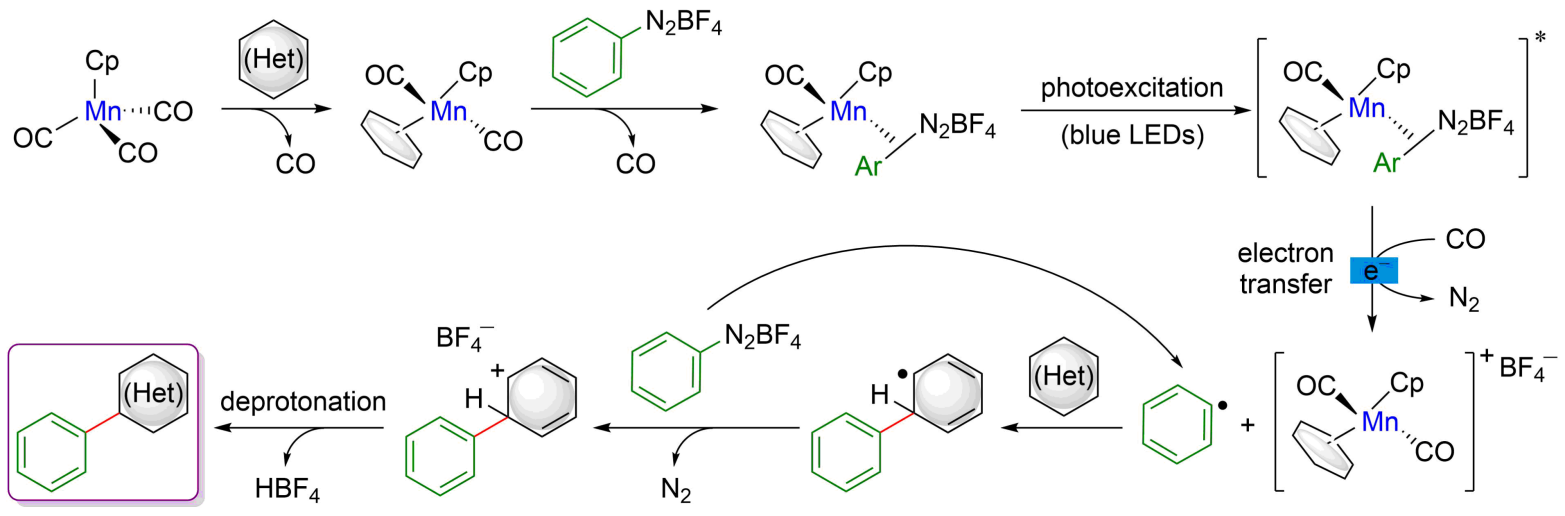
Proposed mechanism for the undirected arylation of (hetero)arenes via dual catalysis.

The mechanistic studies suggested a unique mode of action of this catalytic system (Figure 45). In the initial step both aromatic coupling partners coordinate to the Mn catalyst, thus generating a photoactive complex. Visible-light photoexcitation of the Ar–Mn species and subsequent electron transfer generate the aryl radical together with a cationic Mn species. The addition of the aryl radical to the (hetero)aromatic substrate affords a radical biaryl intermediate that provides the expected coupling product after oxidation and deprotonation.

#### C–H activation of heterocyclic substrates

The panel of C–H bond functionalization reactions encompasses also direct diversification of heteroaromatic compounds. Aromatic heterocycles are key molecular motifs in natural products and biologically active compounds and thus, the development of synthetic methods allowing their site-selective C–H functionalization have focused major attention of the scientific community [13,22]. However, these transformations frequently required noble metals, such as Pd, Rh and Ru, and rather harsh reaction conditions.

**Figure 46:**
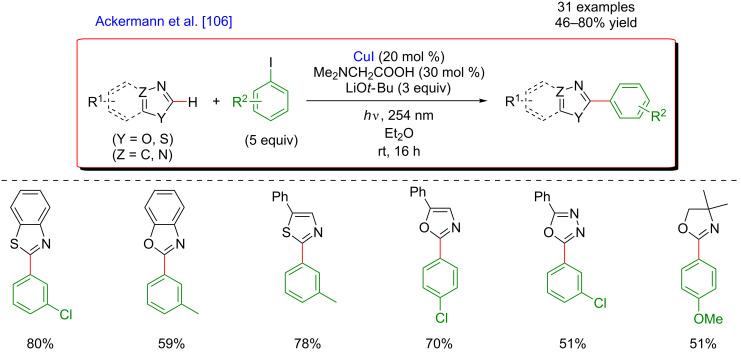
Photoinduced C–H arylation of azoles via copper catalysis.

In 2016, Ackermann reported a clear advance towards more sustainable and milder C–H functionalization of azoles. His research group discovered that the abundant and inexpensive CuI catalyst allowed the direct arylation of benzoxazoles under UV-photoactivation (Figure 46) [106]. Remarkably, this totally site-selective photoinduced C–H arylation took place at room temperature and the use of amino acid ligands allowed a significant improvement of the reaction efficiency. The C–H coupling was compatible with a large panel of heteroaromatic substrates including benzoxazoles, oxazoles, thiazoles, and oxadiazoles, as well as non-aromatic oxazolines. Although the initial protocol necessitated the use of a photoreactor emitting UV light at 254 nm, the same reactivity was also reached under visible-light irradiation (blue LEDs), and in the presence of the photosensitizer, Ir(ppy)_3_. This transformation hence illustrates clearly the potential of combining metal catalysis and photoinduction to design much milder and sustainable C–H transformations.

**Figure 47:**
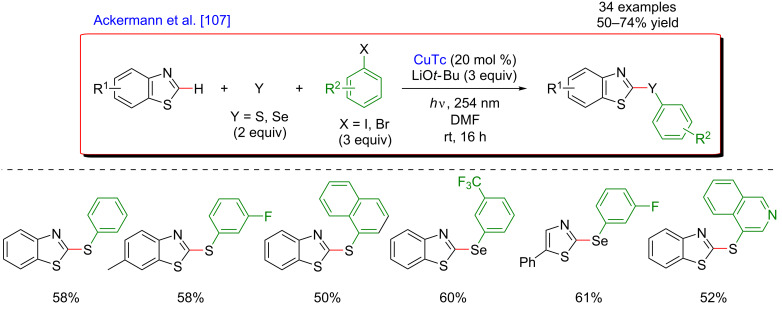
Photo-induced C–H chalcogenation of azoles via copper catalysis.

Subsequently, the concept of the photoinduced C–H functionalization of heteroaromatic compounds was extended towards the direct chalcogenation of azoles (Figure 47) [107]. The Ackermann group discovered that while using a Cu catalyst under photoirradiation, the heteroaryl substrates underwent site-selective thioarylation when employing elemental sulfur and aryl iodides as coupling partners. The reaction tolerated well various aryl iodides, including ones bearing generally sensitive functionalities such as cyano or ester groups, thus delivering a large panel of both electron-rich and electro-poor thioarylated heteroaromatic products. Besides, this photo-assisted C–H functionalization was equally efficient when using elemental selenium, hence affording a variety of 2-arylselanylbenzothiazoles.

**Figure 48:**
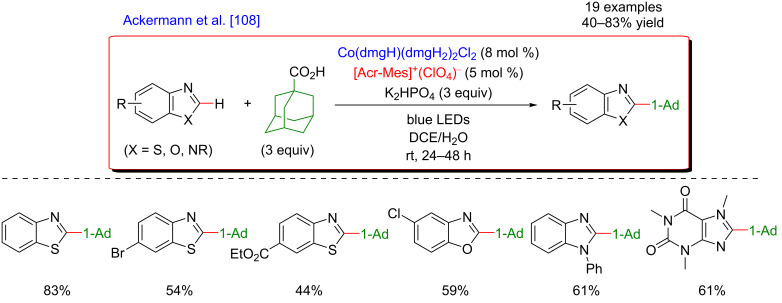
Decarboxylative C–H adamantylation of azoles via dual cobalt/photoredox catalysis.

In 2019, Ackermann et al. demonstrated that a mild and direct C–H functionalization of azoles now was also achieved using an easily accessible cobaloxime catalyst in combination with visible-light irradiation (458 nm), harvested by an organic photosensitizer, an acridinium salt (Figure 48) [108]. Such a dual catalytic system enhanced the decarboxylative C–H adamantylation of heterocycles to occur at room temperature. Thanks to the compatibility of this protocol with various substituted azoles and more complex heterocycles such as benzimidazole, and caffeine derivatives, the strategy is now an appealing procedure to incorporate adamantyl scaffolds.

**Figure 49:**
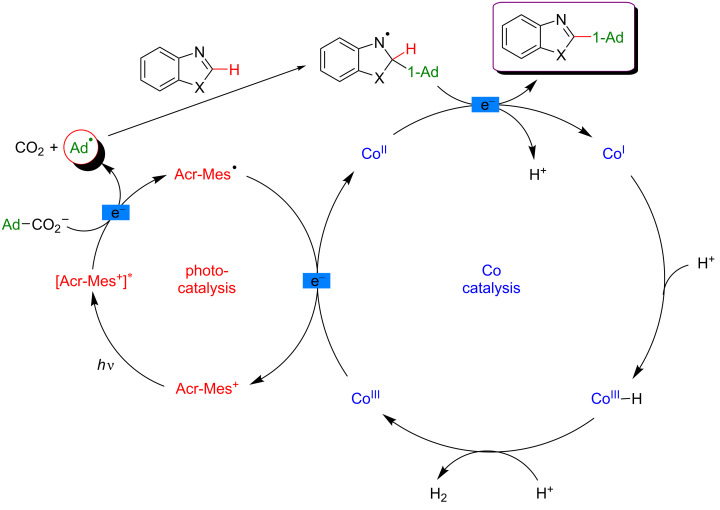
Proposed mechanism for the C–H adamantylation of azoles via dual catalysis.

The proposed mechanism of this transformation combines two catalytic cycles (Figure 49). The photocatalytic transformation implies the irradiation of the photosensitizer and oxidative decarboxylation of the adamantylcarboxylate substrate, delivering an adamantyl radical. The attack of the latter at the electrophilic C2 position of the benzothiazole generates a nitrogen-centered radical intermediate. Subsequent deprotonation of this intermediate and concomitant SET reduction of Co(II) into Co(I) delivers the functionalized product, while the Co(I) complex is believed to capture a proton. The resulting Co(III) species, after the release of H_2_, is finally reduced to the active Co(II) catalyst via SET with concomitant oxidation of the Acr-Mes^•^ radical to the Acr-Mes^+^ ground-state photosensitizer.

#### Modern Sonogashira-type reactions

**Figure 50:**
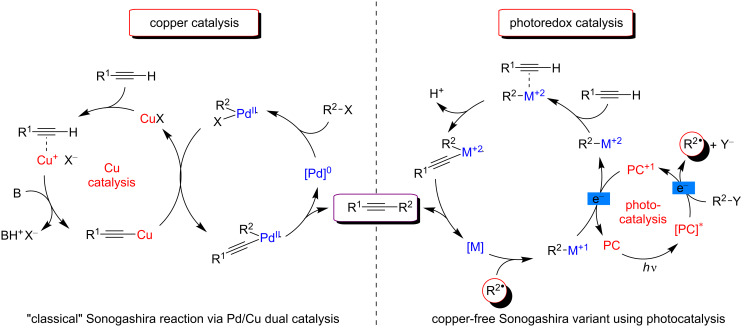
General mechanisms for the “classical” (left) and Cu-free variant (right) Sonogoshira reaction.

In traditional Sonogashira cross-coupling reactions, a catalytic amount of a transition metal (generally Pd) is used in combination with a copper salt co-catalyst. This way, the classical Sonogashira reaction promotes the C–C bond formation between terminal alkynes and aryl (or vinyl) halides via a dual Pd/Cu catalysis [109–110]. The general mechanism of this initially reported C–H functionalization protocol is illustrated in Figure 50 (left). The active Pd(0) catalyst undergoes oxidative addition with the aryl or vinyl halide coupling partner to furnish a Pd(II) species. In parallel, regarding the copper catalytic cycle, a π–alkyne complex is formed thanks to the presence of a base. This results in an increased acidity of the proton of the terminal alkyne substrate, and upon deprotonation, a copper acetylide is generated. The acetylide then reacts with the Pd(II) complex in a transmetalation step, producing an alkynyl Pd(II) intermediate and regenerating the copper catalyst. Finally, the reductive elimination yields the desired coupling product along with the regeneration of the active Pd catalytic species.

While the presence of copper salts was valuable for the efficiency of the transformation, their use in the "classical" Sonogashira cross couplings resulted in several drawbacks. Indeed, copper salts led to the generation of unwanted alkyne homocoupling side products and the requirement of rigorous inert atmosphere. Therefore, in order to exclude copper from the reaction mixture, significant attention was focused on the development of alternative Cu-free Sonogashira reactions [111–112]. In that respect, a possible variant relied on the use of a photoredox catalysis in combination with transition-metal catalysis (Figure 50, right). The mechanism of such a dual catalytic system is initiated by the generation of aryl radicals via SET from an excited photosensitizer to an appropriate radical precursor substrate. Such aryl radical reacts with a transition-metal catalyst (M), resulting in the generation of an oxidized M(+1) complex intermediate. An additional SET event with the photocatalyst thus allows the formation of a M(+2) oxidation state species. The subsequent formation of a σ-bonded alkynyl–M(+2) complex upon deprotonation allows final reductive elimination, delivering the expected coupling product along with the regenerated metal catalyst M. The overall process thus allowed the replacement of copper salts with a suitable photocatalyst.

**Figure 51:**
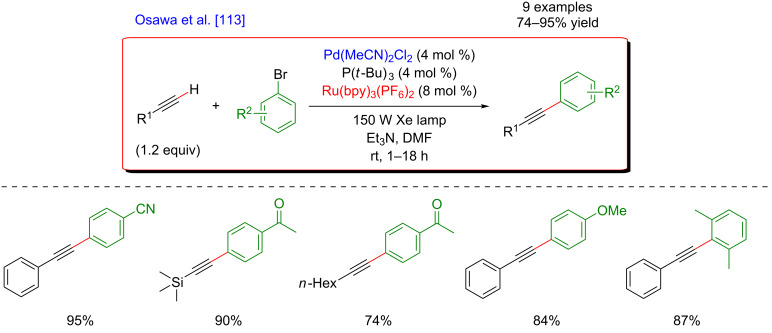
First example of a dual palladium/photoredox catalysis for Sonogashira-type couplings.

The first example of such a dual catalytic system combining photoredox catalysis and palladium catalysis was reported in 2007 by Osawa’s group [113]. The authors developed a Cu-free Sonogashira coupling between aryl halides and terminal alkynes (Figure 51). The efficiency of this transformation was clearly improved by visible-light irradiation of the reaction medium in the presence of a ruthenium-based photocatalyst, although the role of the latter was not fully elucidated (traces of the coupling product formed when performing the reaction in the dark).

**Figure 52:**
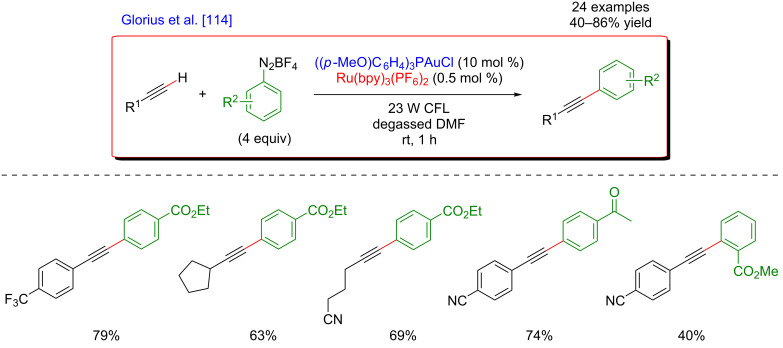
Arylation of terminal alkynes with diazonium salts via dual gold/photoredox catalysis.

More recently, an alternative protocol for a mild Sonogashira-type reaction implying dual catalysis was disclosed by Glorius et al. (Figure 52) [114]. The coupling between terminal alkynes and aryldiazonium salts was achieved at room temperature due to a synergistic system combining gold catalysis and photocatalysis. Under the optimized reaction conditions, merging a Au(I) catalyst and a Ru-based photosensitizer under irradiation with CFL, a large panel of terminal alkynes were coupled with aryldiazonium salts, furnishing the expected arylalkyne products in high yields. Remarkably, this protocol was efficient at room temperature and tolerated well a number of substituents including halogens, motifs frequently incompatible with Pd-based catalytic systems.

**Figure 53:**
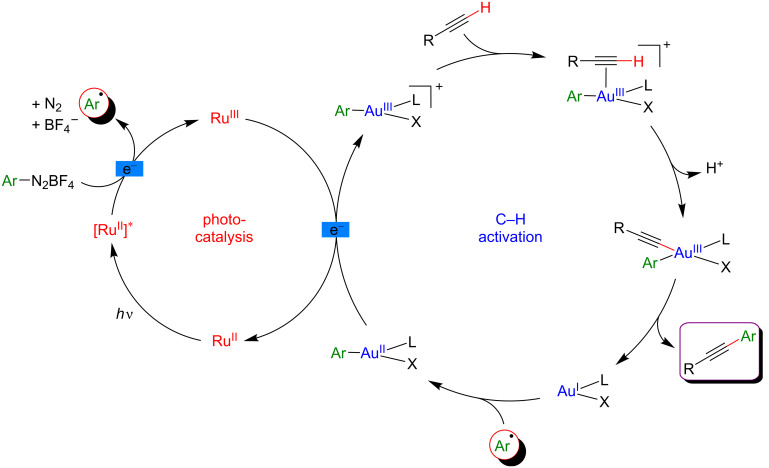
Proposed mechanism for the arylation of terminal alkynes via dual catalysis.

The mechanism of this transformation is initiated by the generation of aryl radicals via a SET process from the excited [Ru(bpy)_3_]^2+^ photocatalyst to the diazonium salt (Figure 53). The thus formed aryl radical then reacts with the Au(I) catalyst, resulting in the generation of an Ar–Au(II) intermediate. A second SET event with the oxidized-photosensitizer (or alternatively with an additional equivalent of aryldiazonium salt) allows the formation of a Au(III) species, featuring high Lewis acidity. The subsequent generation of a σ-bonded alkynyl–Au(III) complex upon deprotonation allows final reductive elimination, delivering the expected coupling product along with the regeneration of the Au(I) catalyst.

#### C–H functionalization by merging visible-light-induced H-abstraction and metal-catalyzed cross-coupling

The field of synergistic metallaphotoredox catalysis for C–H functionalization is also complemented by a conceptually very different approach, based on a combination of hydrogen-atom-transfer (HAT) process and metal-catalyzed cross-couplings. In this transformation, a photoinduced event promotes the generation of species capable to abstract hydrogen from an aliphatic substrate generating an alkyl radical, that is subsequently trapped by a transition-metal catalyst, and thus enabling the cross-coupling-type bond-formation event to take place. This strategy is therefore very complementary to the previously presented ones, in which metal catalysis was generally employed to promote site-selective C–H activation. The formation of distinctive coupling products may thus be targeted.

#### α-Amino and α-ether C(sp^3^)–H functionalization

**Figure 54:**
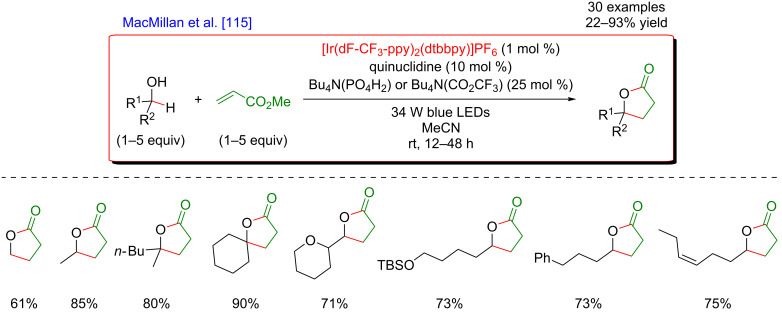
C–H Alkylation of alcohols promoted by H-atom transfer (HAT).

The pioneering and groundbreaking discovery in metallaphotoredox C(sp^3^)–H HAT-type functionalization was reported by MacMillan in 2015 [115]. The development of this transformation was initially inspired by the discovery that strong C–H bonds were homolytically cleaved in a highly selective manner by matching electronic polarity in a C–H bond and an HAT catalyst (Figure 54).

**Figure 55:**
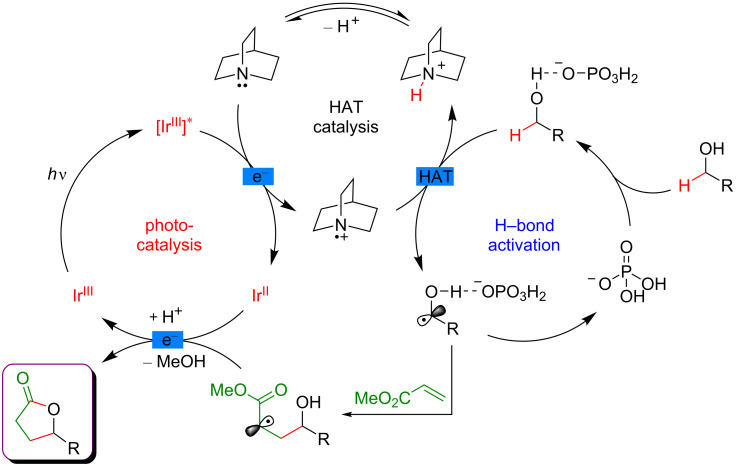
Proposed mechanism for the C–H alkylation of alcohols promoted by HAT.

Indeed, when combining HAT and photoredox catalysis, strong C–H bonds in α position to the hydroxy group motif were selectively cleaved in the presence of weaker and activated C–H bonds. The thus generated α-oxy radicals, after addition to an electron-deficient acrylate, delivered alkyl radicals, that ultimately converted into the lactone-type products (Figure 55).

**Figure 56:**
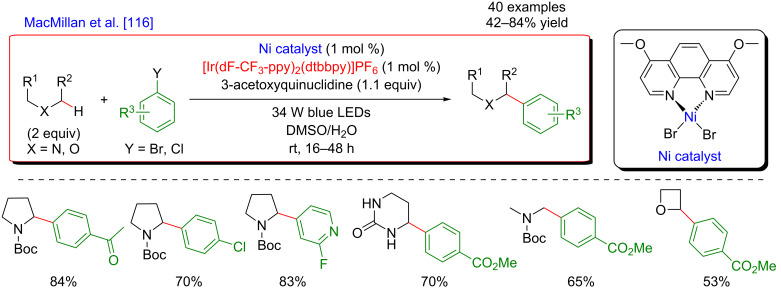
C(sp^3^)–H arylation of latent nucleophiles promoted by H-atom transfer.

Based on this seminal work, the concept of polarity-match HAT selective C(sp^3^)–H abstraction was subsequently used in combination with transition-metal catalysis. When combining a photoredox catalytic cycle (using an Ir(III)-based photosensitizer) with an organocatalytic cycle (allowing SET oxidation of quinuclidine, and subsequent HAT activation of amines), an α-amino C(sp^3^)–H hydrogen abstraction smoothly occurred, delivering radical species, as competent coupling partners in Ni-catalyzed arylation reactions [116]. Remarkably, this complex catalytic system embracing three catalytic cycles was very general, allowing the α-arylation of not only diverse amines (including cyclic and acyclic ones), but also of ethers and amides. Besides, the reaction well tolerated various aryl bromides and aryl chlorides as coupling partners, including aromatics bearing challenging functional groups (sulfonyl and ester), and heterocycles. The perfect orchestration of these three catalytic cycles warranted high efficiency of the coupling under mild reaction conditions (Figure 56).

**Figure 57:**
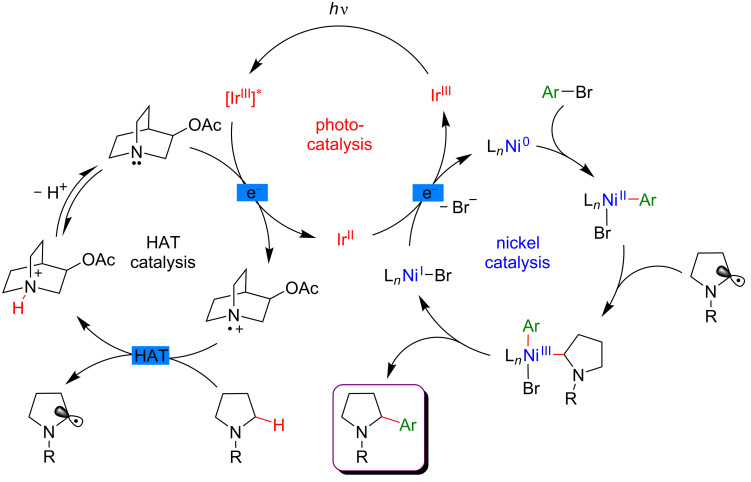
Proposed mechanism for the C(sp^3^)–H arylation of latent nucleophiles promoted by HAT.

The mechanism of this reaction starts by the excitation of the Ir(III) photocatalyst, furnishing a long-lived photoexcited state. SET between the *Ir(III) and 3-acetoxyquinuclidine (a tertiary amine HAT catalyst) generates an Ir(II) species together with key radical amine cation (Figure 57). This finely designed radical amine cation needs to be sufficiently electron-deficient to engender a kinetically selective HAT event at the most electron-rich site of the C–H substrate (polarity matching), thus affording the aliphatic α-amino radical. Of note is that this abstraction event is coherent with thermodynamic considerations, as the bond-dissociation energies (BDEs) of hydridic α-amino C–H bonds are lower than the N–H bond of quinuclidinium (C–H bond = 89 to 94 kcal/mol vs H–N^+^ bond = 100 kcal/mol). In parallel, the Ni(II) precatalyst undergoes double SET reductions by the Ir PC to generate the catalytically active Ni(0) species. Oxidative addition of Ar–Br then furnishes the electrophilic Ni(II)–aryl intermediate, rapidly intercepting the α-amino radical. The Ni(III)–aryl–alkyl complex dispenses the coupling product via reductive elimination and a Ni(I) complex that is ready to undergo SET reduction thus re-engaging in a new catalytic cycle.

**Figure 58:**
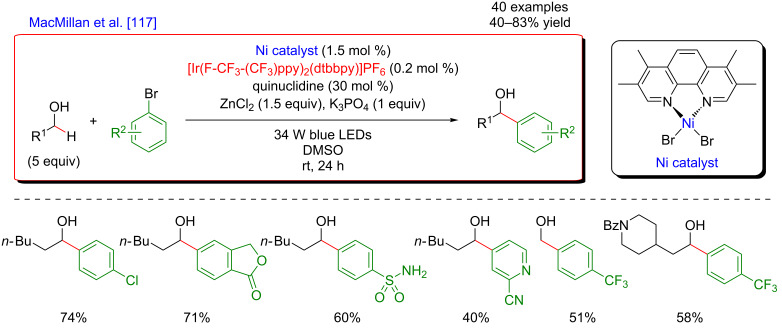
Direct α-arylation of alcohols promoted by H-atom transfer.

The concept of the selective generation of active aliphatic coupling partners by means of H-abstraction through polarity-induced effects and promotion of the C–C-bond formation via a combination of photo- and Ni catalysis also paved the way towards a new approach for the synthesis of benzylic alcohols, important scaffolds in medicinal chemistry [117]. MacMillan discovered that when submitting alcohols to the conditions promoting the HAT-type process, a chemoselective generation of α-C–OH carbon-centered radicals took place while suppressing the formation of the expected O-centered radicals. This perfectly designed catalytic system was very general and compatible with a diversity of aliphatic alcohols, including methanol, and various cyclic ones. Of note is also the particular chemoselectivity of this reaction, as the α-C–OH carbon-centered radicals were preferentially formed over α-C–OR and α-C–N radicals. In addition, diversely substituted aryl bromides and bromopyridine derivatives could be used as the coupling partners. The application of this methodology for the expedient 3-step Prozac’s synthesis clearly highlights the potential of this methodology (Figure 58).

**Figure 59:**
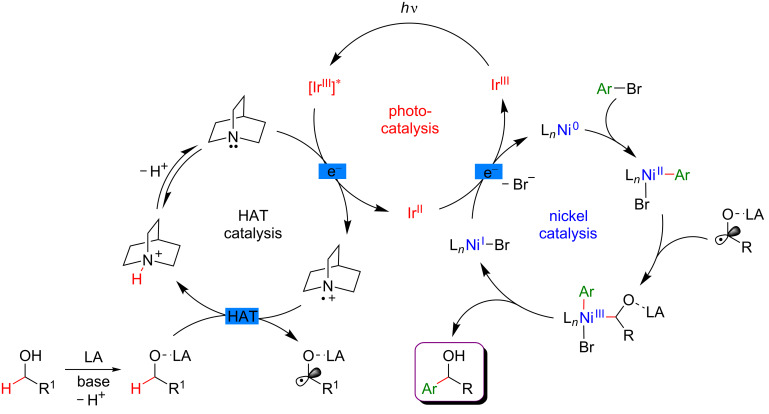
Proposed mechanism for the direct α-arylation of alcohols promoted by HAT.

The addition of a Lewis acid (LA) is the key to success to trigger this uncommon reactivity as metal alkoxide species are formed, exhibiting greatly exchanged hydric character at the α-alkoxy C–H positions (Figure 59). The optimization study showed that ZnCl_2_ was the optimal Lewis acid while quinuclidine played efficiently the role of the HAT catalyst. The thus generated aliphatic radical could now be engaged in the Ni catalytic cycle; after an oxidative addition of the Ni(0) species to the Ar–Br, the α-alkoxy radical is trapped, generating a Ni(III) species that facilitates the reductive elimination step, thus liberating the coupling product, i.e., the benzylic alcohol. The overall catalytic system is complemented with a photocatalytic cycle: the photoexcited Ir(III) photocatalyst promotes the oxidation of the HAT catalyst quinuclidine while the regeneration of the Ir(III) catalyst from Ir(II) warrants the efficient reduction of the Ni(I) intermediates into the catalytically active Ni(0) species.

**Figure 60:**
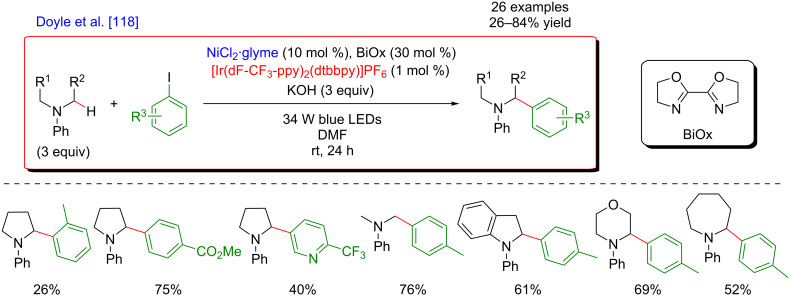
C–H arylation of amines via dual Ni/photoredox catalysis.

Regarding the major advantage of this approach, i.e., a possibility to selectively cleave a strong C–H bond while avoiding functionalization of other, usually more reactive positions, it was not surprising that this strategy has been investigated further. Therefore, a complementary protocol for the α-arylation of arylamines was disclosed by Doyle (Figure 60) [118].

**Figure 61:**
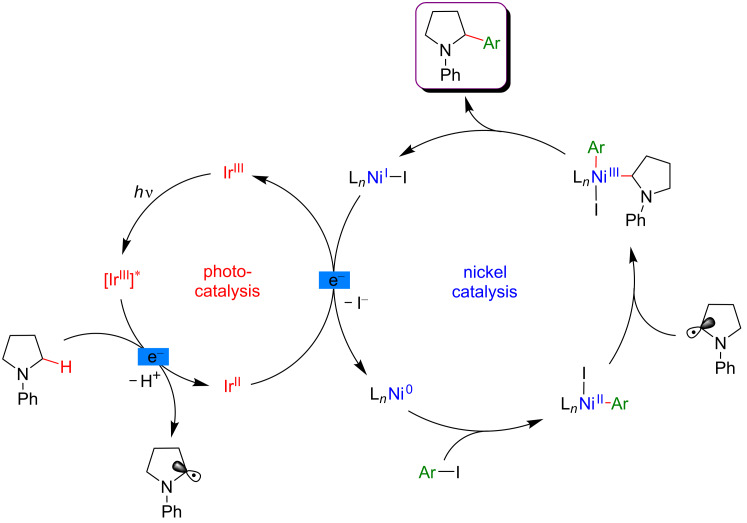
Proposed mechanism for the C–H arylation of amines via dual Ni/photoredox catalysis.

In this case, an α-amino radical is believed to be directly generated via a SET process with the photoexcited Ir(III) catalyst (Figure 61). In analogy to the system developed by MacMillan, the Ni catalytic cycle implies the oxidative addition of Ar–I to an in situ generated Ni(0) species delivering the Ni(II)–aryl intermediate, followed by trapping of the radical coupling partner. The expected C–C-bond formation occurring during the reductive elimination step furnishes the α-arylated amines. Of note is that this modified protocol, using a Ni complex coordinated with the BiOx ligand, KOH as a base, and DMF as solvent, was compatible with *N*-arylated amines, whereas *N*-Boc-protected substrates were generally used by MacMillan.

**Figure 62:**
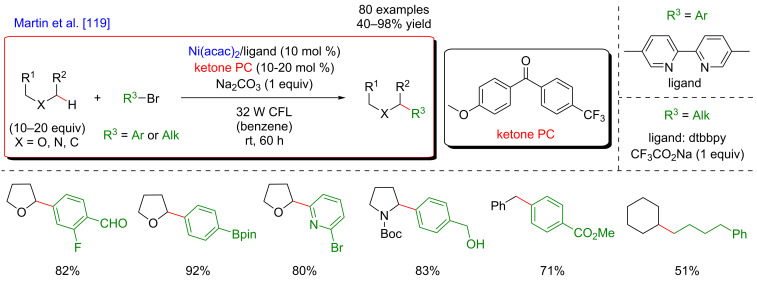
C–H functionalization of nucleophiles via excited ketone/nickel dual catalysis.

In 2018, a major advance in this field was disclosed by Martin [119]. The author hypothesized that a carefully selected triplet ketone sensitizer should be able, under light activation, to promote the HAT and SET steps, thus allowing both, hydrogen abstraction in the α-position to a heteroatom and reduction of the Ni(I) species. Accordingly, a direct functionalization of feedstock alkanes (such as THF) was achieved in the absence of a costly Ir-based photosensitizer. Following the initial hypothesis, the diarylketone was precisely selected with respect to its absorption in the visible-light range. The Ni catalyst was also supported with a bipyridine-type ligand, and under the optimized reaction conditions, the direct C(sp^3^)–H arylation, and alkylation of THF were achieved smoothly (Figure 62). The protocol turned out to be highly tolerant with respect to various aromatic bromines, including *ortho*-substituted ones as well as aromatics bearing complex motifs, and bromoalkanes, even in the presence of a catalytic amount of the diarylketone photosensitizer. Besides, the reactivity was also efficiently extended towards the functionalization of ethers and benzylic C–H bonds.

**Figure 63:**
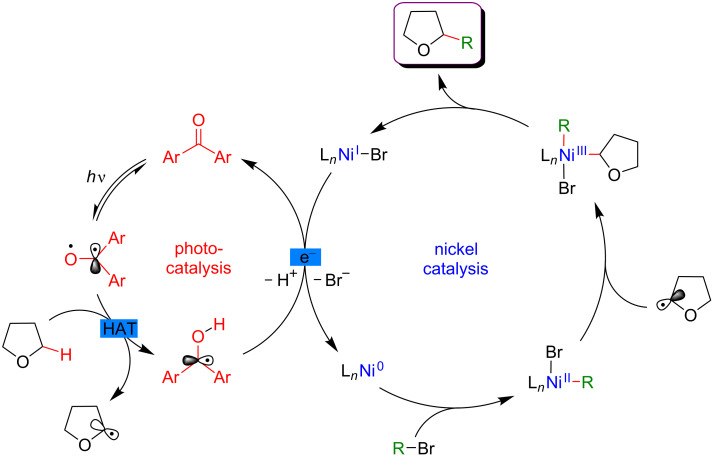
Proposed mechanism for the C–H functionalization enabled by excited ketones.

The experimental mechanistic studies confirmed the initial working hypothesis, evoking the HAT process from the triplet excited state of the diarylketone delivering the key aliphatic radical, followed by SET to recover back the initial form of the diarylketone, with the concomitant generation of the catalytically active Ni(0) catalyst (Figure 63).

**Figure 64:**
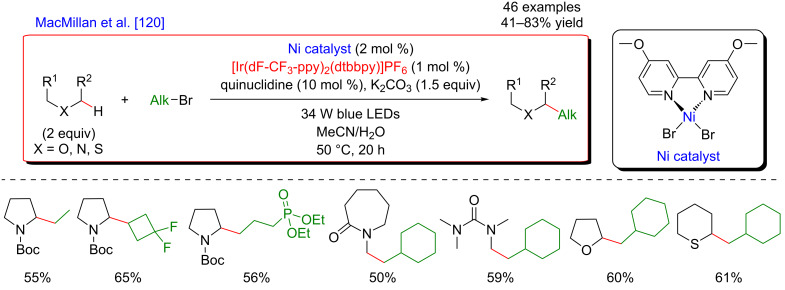
Selective sp^3^–sp^3^ cross-coupling promoted by H-atom transfer.

In 2017, the McMillan group applied the HAT approach to perform sp^3^–sp^3^ coupling. In this case, the polarity-match HAT process was combined with a Ni-catalyzed alkylation [120]. Following a comparable mechanistic scenario orchestrating three catalytic cycles (photoredox, organocatalytic, and Ni-based catalysis), aliphatic radicals in α-position to N, O, and S-atoms were generated and coupled with a large panel of alkyl bromides (both secondary and primary), thus delivering the aliphatic products in high yields and under very mild reaction conditions (Figure 64).

**Figure 65:**
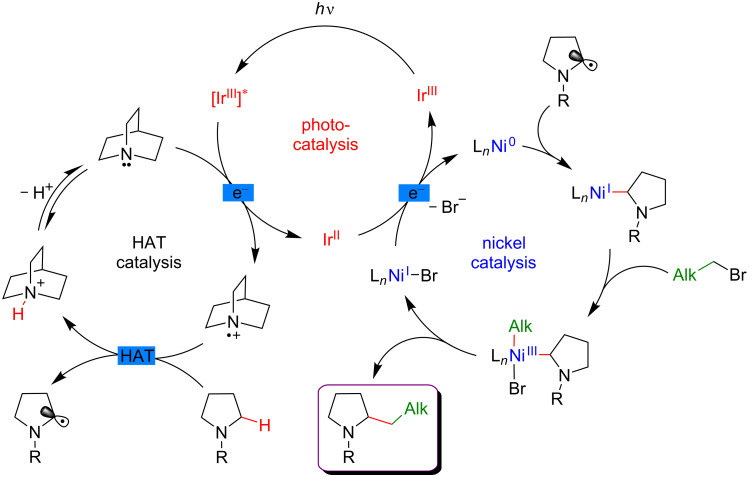
Proposed mechanism for the selective sp^3^–sp^3^ cross-coupling promoted by HAT.

In contrast to the direct arylation reactions, in this sp^3^–sp^3^ coupling, the in situ generated catalytically active Ni(0) complex was believed to trap directly the alkyl radical, prior to the oxidative addition of the alkyl bromide (Figure 65).

**Figure 66:**
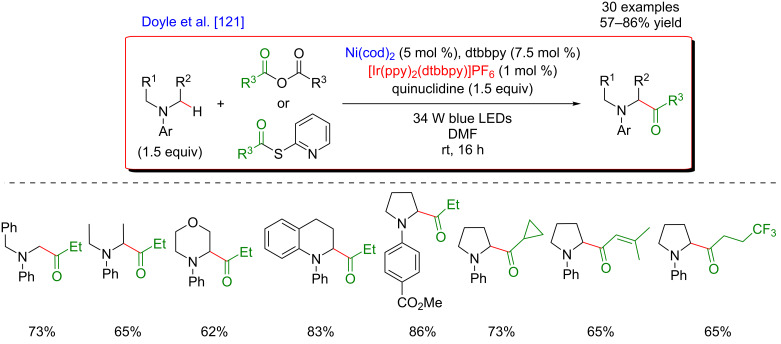
Direct C(sp^3^)–H acylation of amines via dual Ni/photoredox catalysis.

The photocatalytic generation of α-amino radicals was also astutely combined with the Ni-catalyzed acylation reaction. In 2016, Doyle reported that a complex catalytic cycle combining an Ir(III) photosensitizer with a Ni(dtbbpy) catalyst and quinuclidine as base promoted, under blue LED irradiation, the direct coupling of arylamines and anhydrides, delivering a large panel of α-acetylated compounds in good yields (Figure 66) [121].

**Figure 67:**
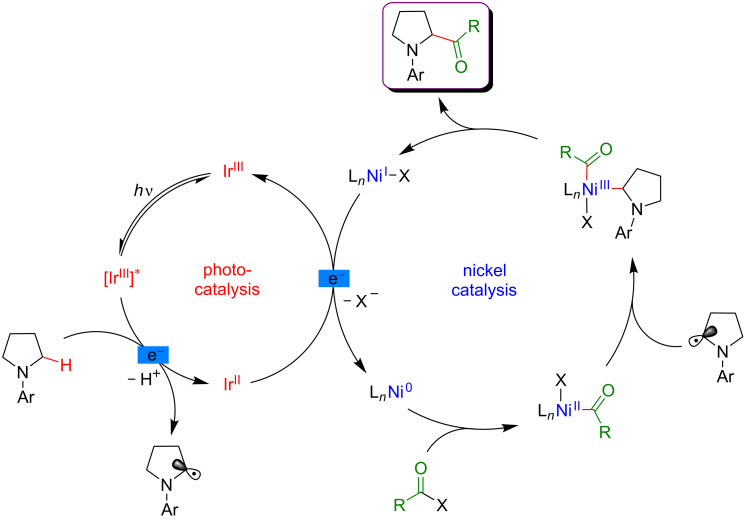
Proposed mechanism for the C–H acylation of amines via dual Ni/photoredox catalysis.

In this case, the Ni(0) active catalyst undergoes an oxidative addition into the acyl precursors and the thus generated Ni(II) complex intercepts an α-amino radical. The resulting Ni(III) species then promotes the rapid reductive elimination while forging the C–C bond formation. The authors suggested the formation of the α-amino radical from SET between the excited *Ir(III) photocatalyst and *N*-phenylpyrrolidine, followed by a deprotonation event. However, as quinuclidine was required in this transformation, a potential HAT-type reactivity could not be excluded (Figure 67).

**Figure 68:**
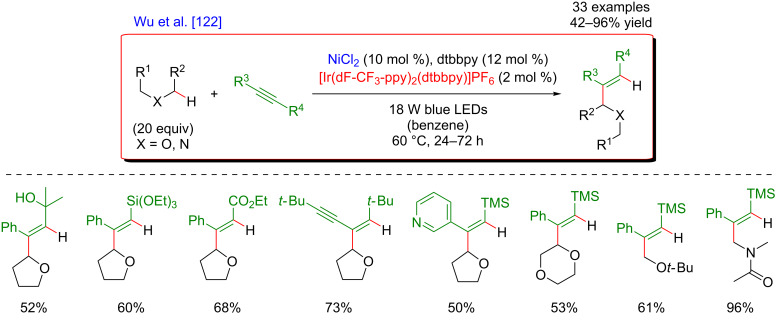
C–H hydroalkylation of internal alkynes via dual Ni/photoredox catalysis.

Recently, the panel of metallaphotoredox C(sp^3^)–H cross-coupling reactions was complemented with the direct alkenylation of ethers and amides. The reaction developed by Wu astutely applied a dual photocatalysis/Ni catalysis to achieve the hydroalkylation of internal alkenes using α-hetero C(sp^3^)–H bond as the coupling partners (Figure 68) [122]. The expected olefinated ethers were obtained in high yields and generally high selectivity using a large panel of alkynes. Of note is that this coupling required an increased reaction temperature (60 °C).

**Figure 69:**
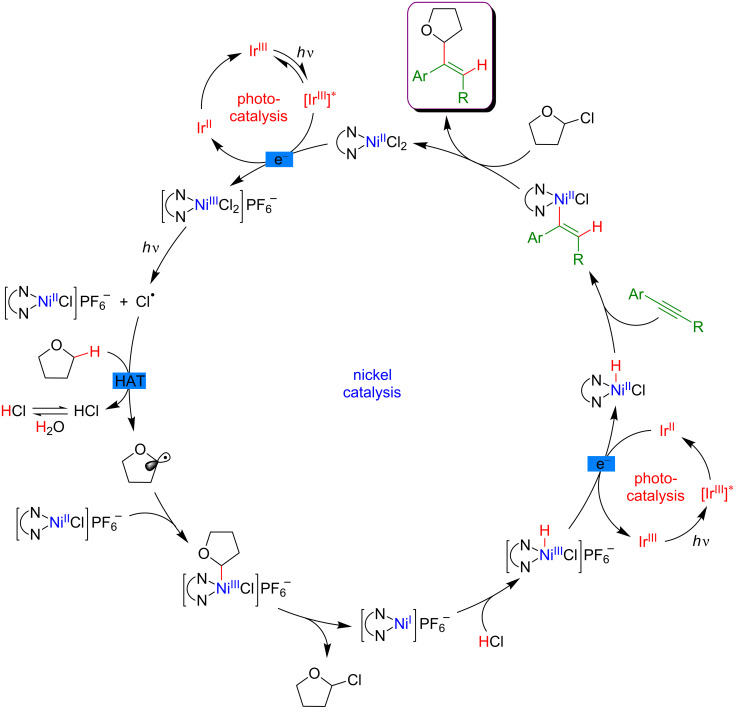
Proposed mechanism for the C–H hydroalkylation of internal alkynes.

From the mechanistic viewpoint, it was speculated that in contrast to other related dual catalytic systems, the photoexcited *Ir(III) complex oxidized the Ni(II)(dtbbpy)Cl_2_ precatalyst to afford a Ni(III) species via SET (Figure 69). Photolysis of the latter Ni(III) species results in a Ni(II) intermediate and a chlorine radical. Hydrogen atom abstraction from an aliphatic substrate thus smoothly occurs in the presence of the chlorine radical, delivering an aliphatic radical that is rapidly trapped by the Ni(II) complex. As the thus generated Ni(III) complex could not undergo carbo-nickelation with alkynes, a reductive elimination is believed to take place delivering a Ni(I) species, together with α-chlorinated THF. The oxidative addition of HCl furnishes the key nickel hydride species. A SET reduction of this intermediate by the oxidation of the Ir(II) species delivers Ni(II) hydride, the active species prompt to undergo the regioselective hydronickelation of the alkyne. The final nucleophilic substitution with the in situ generated chlorinated THF produces the expected functionalized olefin.

**Figure 70:**
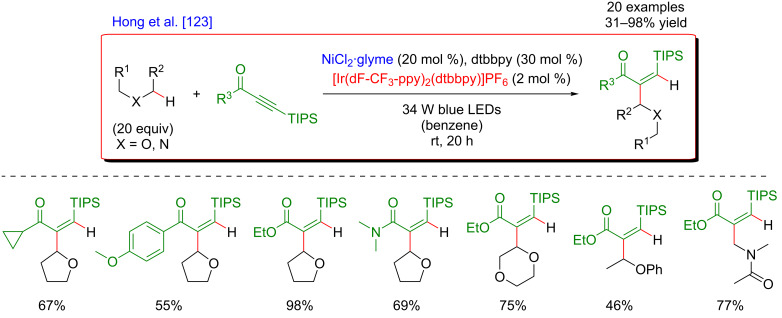
Alternative procedure for the C–H hydroalkylation of ynones, ynoates, and ynamides.

Subsequently, the generality of this C(sp^3^)–H alkenylation was extended towards a similar hydroalkylation of ynones, ynoates, and ynamides (Figure 70) [123].

#### Allylic C–H functionalization

**Figure 71:**
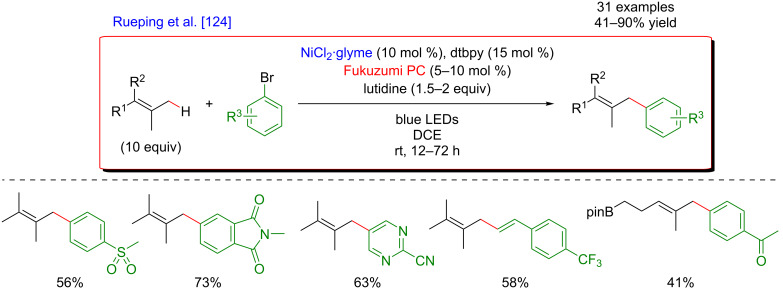
Allylic C(sp^3^)–H activation via dual Ni/photoredox catalysis.

The key step of the H-abstraction by a chlorine radical to achieve C(sp^3^)–H functionalization was further explored by Rueping et al*.* [124]. When reacting tetramethylethylene with aryl bromides under a dual catalytic system including [Acr-Mes]ClO_4_ as photosensitizer, combined with a Ni–dtbpy complex, lutidine as the base, under blue LED irradiation, the formation of vinyl bromides was achieved (Figure 71). Interestingly, this cross-coupling protocol was operative for both, aryl and vinyl bromides, and featured an important group tolerance with respect to the halogenated coupling partners, including heterocyclic motifs.

**Figure 72:**
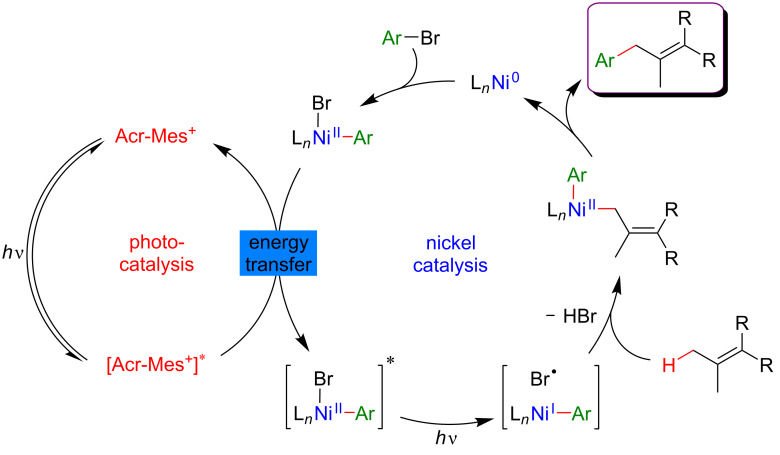
Proposed mechanism for the allylic C(sp^3^)–H activation via dual Ni/photoredox catalysis.

This cross-coupling involves the generation of an allylic radical via abstraction of the hydrogen atom by a bromine radical (Br^•^, resulting from homolysis of the electronically excited Ni(III)–ArBr) which is trapped by the Ni(II)–Ar intermediate, with the reductive elimination step delivering the expected allylic compound (Figure 72).

**Figure 73:**
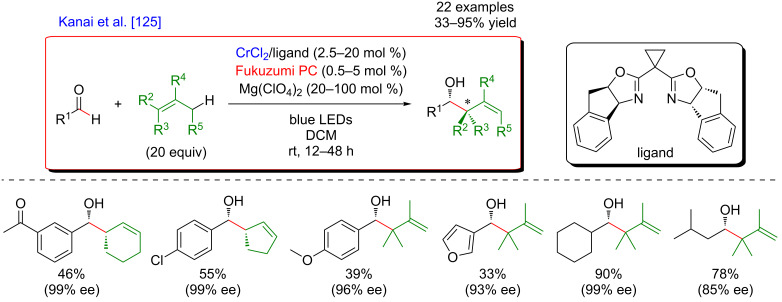
Asymmetric allylation of aldehydes via dual Cr/photoredox catalysis.

Recently, the portfolio of allylic C(sp^3^)–H functionalizations promoted via dual catalysis was extended by merging organophotoredox catalysis and chiral chromium hydride catalysts (Figure 73) [125].

**Figure 74:**
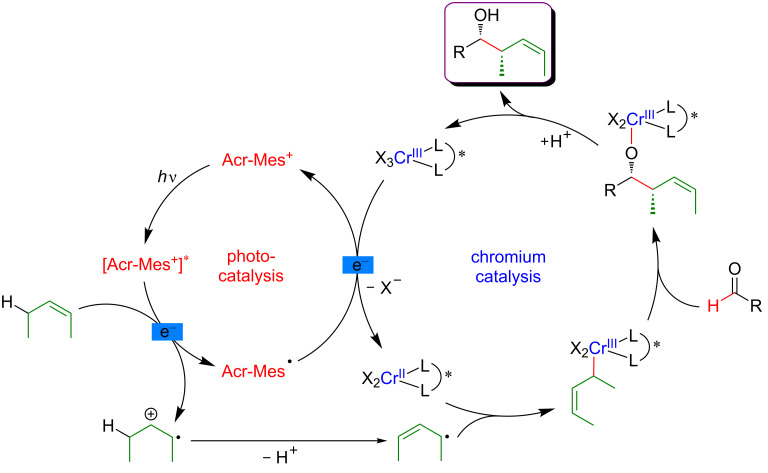
Proposed mechanism for the asymmetric allylation of aldehydes via dual catalysis.

In this case, the allylic radical was expected to be formed via an electron-transfer oxidation of the photoexcited acridinium catalyst. The allyl radicals, trapped by a carefully selected chiral Cr complex thus generate a nucleophilic species ready to engage in an asymmetric allylation of the aldehydes hence furnishing the chiral benzylic alcohols in moderate to good yields, and generally excellent regioselectivities (Figure 74).

#### Functionalization of aldehydes

The direct functionalization of formyl C–H bonds via metal-catalyzed cross-couplings attracts constant scientific attention as this approach allows building up of molecular complexity while respecting atom-economy. However, such reactions generally call for the use of stoichiometric amounts of oxidants or reductants, or the preinstallation of a directing group. Aiming for more sustainable C–H formylations, MacMillan hypothesized that formyl radical generation could be possible via an HAT-process while designing a complex system comprising three catalytic cycles, i.e., a photoredox, nickel, and HAT catalysis, might provide an appealing alternative protocol for the direct synthesis of ketones [126].

**Figure 75:**
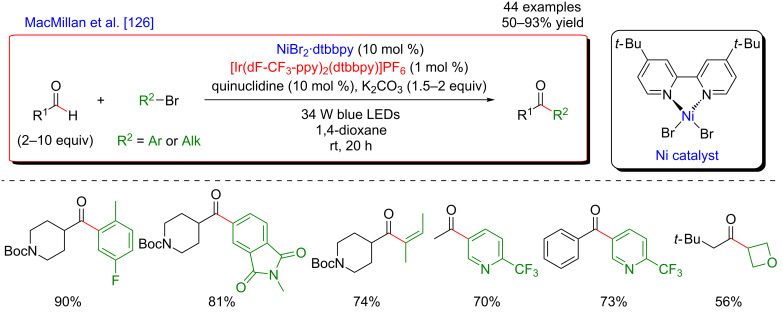
Aldehyde C–H functionalization promoted by H-atom transfer.

In accordance with this working hypothesis, the finely optimized catalytic system including an Ir-based photosensitizer, NiBr_2_-dtbpy complex, quinuclidine, and K_2_CO_3_ base promoted effectively the cross-coupling between diverse aliphatic (cyclic and acyclic) aldehydes, and both, alkyl and aryl bromides (Figure 75). A remarkably large substrate scope could be revealed, delivering the expected ketones in moderate to good yields.

**Figure 76:**
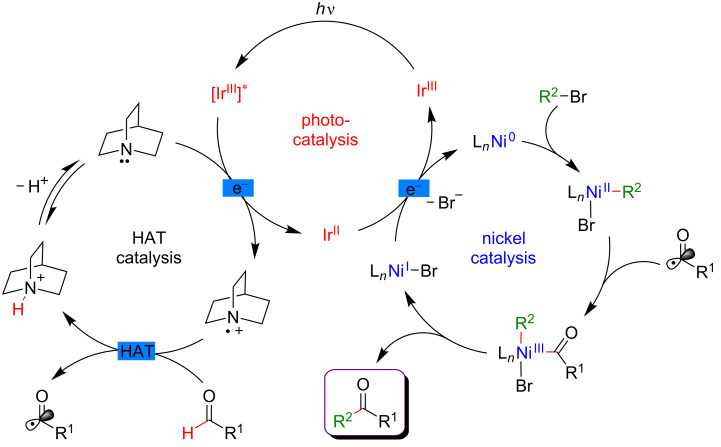
Proposed mechanism for the C–H functionalization of aldehydes promoted by HAT.

The proposed mechanistic scenario evokes initial irradiation of the Ir-based photosensitizer and a subsequent SET oxidation of quinuclidine to form a cationic radical (Figure 76). An HAT between the quinuclidine radical and the aldehyde furnishes the key acyl radical. Both, the hydric and relatively weak character of this bond dictates the selectivity of this C–H bond cleavage event. In parallel, the Ni(I) complex engages with the reduced Ir(II) photosensitizer in an SET-type reaction delivering a Ni(0) species and regenerating the Ir(III) photocatalyst. The oxidative addition of Ni(0) to the Ar–Br bond then delivers the Ar–Ni(II)–Br species able to trap the acyl radical, thus forming a Ni(III)–Ac intermediate. Finally, the reductive elimination liberates the expected ketone while regenerating the Ni(I) complex.

#### Strong, neutral C–H bonds

**Figure 77:**
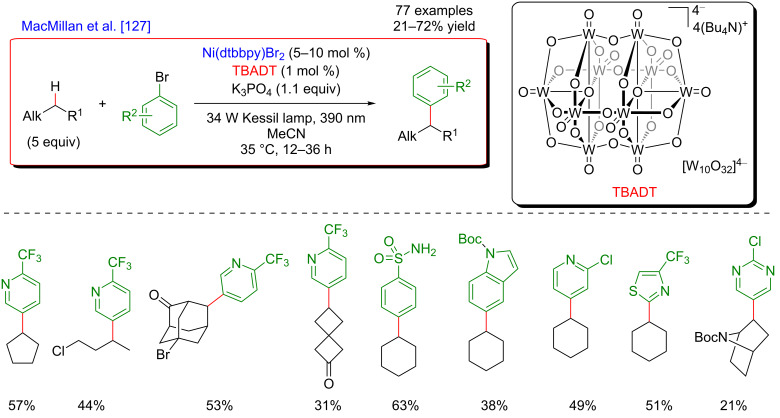
Direct C–H arylation of strong aliphatic bonds promoted by HAT.

In order to complete the panel of metallaphotoredox functionalization of C(sp^3^)–H bonds, the group of MacMillan focused on the design of a catalytic system compatible with simple and non-activated aliphatic substrates. Based on their previous groundbreaking achievements in the HAT type C–H functionalization, they surmised that strong, C–H bond abstraction could be feasible by a catalyst possessing high-energy excited states, such as polyoxometalates (POMs), and in particular the decatungsten anion [W_10_O_32_]^4−^ [127]. Such carbon-centered radicals could thus be rapidly intercepted in the Ni catalytic cycle, to undergo cross-coupling delivering the expected arylated alkanes. As foreseen, while using tetrabutylammonium decatungsten (TBADT) as the HAT catalyst, together with Ni(dtbbpy)Br_2_ as the metallic precursor, the expected cross-coupling occurred smoothly using a Kessil lamp (390 nm). Remarkably, diverse of alkanes were applicable, and the regioselectivity of the reaction was dictated by TBADT, with the H-abstraction occurring preferentially at the less-hindered positions. Accordingly, a very large panel of functionalized products was isolated in good yields (Figure 77). This unique transformation was further extended towards the coupling with heteroaromatic halides and its synthetic potential was illustrated by the late-stage functionalization of a few natural products.

**Figure 78:**
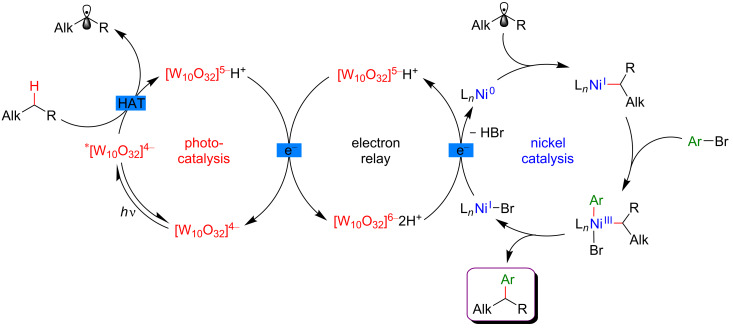
Proposed mechanism for the C–H arylation of strong aliphatic bonds promoted by HAT.

Following the overall mechanistic scenario, the photoexcitation of tetrabutylammonium decatungsten (TBADT) and subsequent intersystem crossing lead to the triplet excited state capable of abstract a H^•^ from the alkyl nucleophile (Figure 78). Deprotonation of the single reduced TBADT regenerates the HAT active [W_10_O_32_]^4−^. The in situ reduction of the Ni(II) precatalyst delivers the catalytically active Ni(0) intermediate, rapidly intercepting the carbon-centered radical. Final oxidative addition of the thus generated alkyl-radical intermediate into the Ar–X bond furnishes a Ni(III) species, rapidly liberating the expected final product via a reductive elimination event.

**Figure 79:**
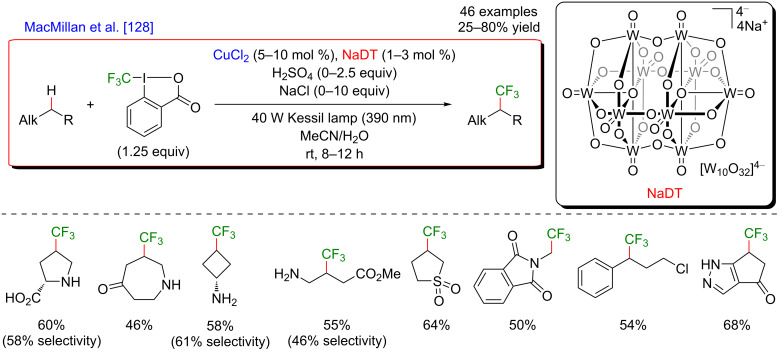
Direct C–H trifluoromethylation of strong aliphatic bonds promoted by HAT.

More recently, the decatungstate-catalyzed HAT C(sp^3^)–H abstraction was also combined with Cu to promote aliphatic trifluoromethylation [128]. As in the previous example, the site-selectivity of the H-abstraction step was directed into the most sterically accessible, electron-rich C(sp^3^)–H bond. This unique and extremely broad methodology gave, therefore, access to original trifluoromethylated amines in general moderate to high yields and was perfectly suitable for a selective late-stage functionalization of even sophisticated molecular scaffolds, including drugs (Figure 79).

**Figure 80:**
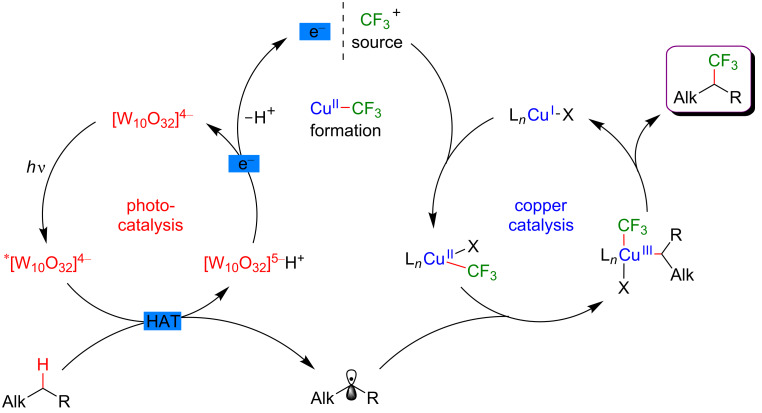
Proposed mechanism for the C–H trifluoromethylation of strong aliphatic bonds.

In parallel, the interception of a CF_3_^+^ species (generated via formal reduction of the Togni II reagent by the decatungstate reduced species) by a Cu(I) species followed by trapping of the HAT-generated alkyl radial, allow the formation of the key Cu(III)–alkyl–CF_3_ intermediate. Upon a reductive elimination step, the trifluoromethylated products were thus obtained (Figure 80).

## Conclusion

Transition metal-catalyzed C–H bond activation and visible-light photocatalysis are amongst the most rapidly expanding fields in organic chemistry and these approaches display complementary characteristics and applications. Therefore, it is not surprising that over the last decade, the development of dual catalytic systems, combining these two different strategies in a single chemical transformation has attracted a growing attention of the scientific community. Initially, a visible-light photoredox cycle was used as a modern and astute solution to reoxidize the metal catalysts in various direct C–H functionalization reactions, thus obviating the need for a stoichiometric amount of an external oxidant. This approach permitted designing more ecofriendly and sustainable alternatives in comparison with “classical” C–H-activation reactions. Progressively, a second-generation of a “dual synergistic catalysis” system was disclosed. In such complex catalytic systems, both C–H activation and photocatalysis are used as independent but complementary activation modes, allowing the generation of two distinct reactive species, whose recombination furnishes coupling products under generally mild reaction conditions. Such synergistic dual catalysis gives thus a promise of devising previously inaccessible transformations and the synthesis of coupling products difficult to access via a traditional C–H activation strategy may be envisioned. Although this approach seems particularly tempting, the reported examples of such protocols still remain rare. The inherent complexity of this dual catalysis revolves around the fine orchestration of the two or even three distinct catalytic cycles acting in a cooperative way to successfully promote the formation of a new bond. Indeed, the design of catalytic conditions in adequation, perfectly compatible with both independent cycles and promoting the generation of reactive intermediates with comparable kinetics, is far from trivial.

Very recently, procedures in which a C–H activation catalytic cycle affords in situ generations of photoactive metallacyclic intermediates, prompt to harvest the light and trigger subsequent catalytic steps, were disclosed. This metallaphotoredox catalysis, occurring under exogenous photosensitizer-free conditions, opens the way towards very original and environmentally benign transformations.

In parallel, a complementary strategy emerged, in which HAT-type processes were used to generate aliphatic radicals via selective C–H abstraction. Such aliphatic radicals turned out to be prominent coupling partners in Ni, and more recently, Cu cross-couplings, thus unlocking the door towards highly challenging C(sp^3^)–C(sp^2^), C(sp^3^)–C(sp^3^), and C(sp^3^)–CF_3_ couplings. Importantly, this approach allows the selective functionalization of strong C–H bonds, which is difficult to achieve via alternative routes.

Accordingly, such dual catalysis opens new perspectives in the field of organic synthesis. Certainly, despite the intrinsic difficulty in designing complex catalytic systems, many efforts will be concentrated on conceptualizing alternative modes of merging C–H bond activations and visible-light photocatalysis. In this way, the development of unique and unprecedented transformations can be expected, thus providing new solutions for sustainable and eco-responsible synthesis. Besides, mild reaction conditions, limited waste generation, and rapidly spreading implementation of photocatalysis render these coupling reactions of immense interest for industrial applications. In particular, the development of flow-type protocols for such dual catalysis may be foreseen, thus further increasing the synthetic potential of these reactions. Attention will therefore probably be focused on the general use of cheap, organic photocatalysts capable to harvest efficiently visible light.

## References

[1] Labinger J A, Bercaw J E (2002). Nature.

[2] Lyons T W, Sanford M S (2010). Chem Rev.

[3] Ackermann L (2011). Chem Rev.

[4] Wencel-Delord J, Dröge T, Liu F, Glorius F (2011). Chem Soc Rev.

[5] Yeung C S, Dong V M (2011). Chem Rev.

[6] Song G, Wang F, Li X (2012). Chem Soc Rev.

[7] Arockiam P B, Bruneau C, Dixneuf P H (2012). Chem Rev.

[8] Rouquet G, Chatani N (2013). Angew Chem, Int Ed.

[9] Chen Z, Wang B, Zhang J, Yu W, Liu Z, Zhang Y (2015). Org Chem Front.

[10] Gensch T, Hopkinson M N, Glorius F, Wencel-Delord J (2016). Chem Soc Rev.

[11] Davies H M L, Morton D (2016). J Org Chem.

[12] Wei Y, Hu P, Zhang M, Su W (2017). Chem Rev.

[13] Yang Y, Lan J, You J (2017). Chem Rev.

[14] Gandeepan P, Müller T, Zell D, Cera G, Warratz S, Ackermann L (2019). Chem Rev.

[15] McMurray L, O'Hara F, Gaunt M J (2011). Chem Soc Rev.

[16] Gutekunst W R, Baran P S (2011). Chem Soc Rev.

[17] Newhouse T, Baran P S (2011). Angew Chem, Int Ed.

[18] Yamaguchi J, Yamaguchi A D, Itami K (2012). Angew Chem, Int Ed.

[19] Wencel-Delord J, Glorius F (2013). Nat Chem.

[20] Karimov R R, Hartwig J F (2018). Angew Chem, Int Ed.

[21] Jazzar R, Hitce J, Renaudat A, Sofack-Kreutzer J, Baudoin O (2010). Chem – Eur J.

[22] Ackermann L (2010). Chem Commun.

[23] Wasa M, Engle K M, Yu J-Q (2010). Isr J Chem.

[24] Baudoin O (2011). Chem Soc Rev.

[25] Li H, Li B-J, Shi Z-J (2011). Catal Sci Technol.

[26] Qiu G, Wu J (2015). Org Chem Front.

[27] He J, Wasa M, Chan K S L, Shao Q, Yu J-Q (2017). Chem Rev.

[28] Evano G, Theunissen C (2019). Angew Chem, Int Ed.

[29] Fagnoni M, Dondi D, Ravelli D, Albini A (2007). Chem Rev.

[30] Zeitler K (2009). Angew Chem, Int Ed.

[31] Narayanam J M R, Stephenson C R J (2011). Chem Soc Rev.

[32] Xuan J, Xiao W-J (2012). Angew Chem, Int Ed.

[33] Shi L, Xia W (2012). Chem Soc Rev.

[34] Tucker J W, Stephenson C R J (2012). J Org Chem.

[35] Reckenthäler M, Griesbeck A G (2013). Adv Synth Catal.

[36] Fukuzumi S, Ohkubo K (2013). Chem Sci.

[37] Xi Y, Yi H, Lei A (2013). Org Biomol Chem.

[38] Prier C K, Rankic D A, MacMillan D W C (2013). Chem Rev.

[39] Shaw M H, Twilton J, MacMillan D W C (2016). J Org Chem.

[40] Romero N A, Nicewicz D A (2016). Chem Rev.

[41] Xie J, Jin H, Hashmi A S K (2017). Chem Soc Rev.

[42] Wang C-S, Dixneuf P H, Soulé J-F (2018). Chem Rev.

[43] Festa A A, Voskressensky L G, Van der Eycken E V (2019). Chem Soc Rev.

[44] Yoon T P, Ischay M A, Du J (2010). Nat Chem.

[45] Schultz D M, Yoon T P (2014). Science.

[46] Strieth-Kalthoff F, James M J, Teders M, Pitzer L, Glorius F (2018). Chem Soc Rev.

[47] Allen A E, MacMillan D W C (2012). Chem Sci.

[48] Shibasaki M, Kanai M, Matsunaga S, Kumagai N (2009). Acc Chem Res.

[49] Park Y J, Park J-W, Jun C-H (2008). Acc Chem Res.

[50] Xu H, Zuend S J, Woll M G, Tao Y, Jacobsen E N (2010). Science.

[51] Han Z-Y, Xiao H, Chen X-H, Gong L-Z (2009). J Am Chem Soc.

[52] Lathrop S P, Rovis T (2009). J Am Chem Soc.

[53] Hopkinson M N, Sahoo B, Li J-L, Glorius F (2014). Chem – Eur J.

[54] Xie J, Jin H, Xu P, Zhu C (2014). Tetrahedron Lett.

[55] Skubi K L, Blum T R, Yoon T P (2016). Chem Rev.

[56] Lang X, Zhao J, Chen X (2016). Chem Soc Rev.

[57] Ravelli D, Protti S, Fagnoni M (2016). Chem Rev.

[58] Prier C K, MacMillan D W C, Stephenson C R J, Yoon T, MacMillan D W C (2018). Dual Photoredox Catalysis: The Merger of Photoredox Catalysis with Other Catalytic Activation Modes. Visible Light Photocatalysis in Organic Chemistry.

[59] Levin M D, Kim S, Toste F D (2016). ACS Cent Sci.

[60] Fabry D C, Rueping M (2016). Acc Chem Res.

[61] Zhou W-J, Zhang Y-H, Gui Y-Y, Sun L, Yu D-G (2018). Synthesis.

[62] Twilton J, Le C, Zhang P, Shaw M H, Evans R W, MacMillan D W C (2017). Nat Rev Chem.

[63] McLean E B, Lee A-L (2018). Tetrahedron.

[64] Dwivedi V, Kalsi D, Sundararaju B (2019). ChemCatChem.

[65] De Abreu M, Belmont P, Brachet E (2020). Eur J Org Chem.

[66] Tellis J C, Kelly C B, Primer D N, Jouffroy M, Patel N R, Molander G A (2016). Acc Chem Res.

[67] Gui Y-Y, Sun L, Lu Z-P, Yu D-G (2016). Org Chem Front.

[68] Zuo Z, Ahneman D T, Chu L, Terrett J A, Doyle A G, MacMillan D W C (2014). Science.

[69] Tellis J C, Primer D N, Molander G A (2014). Science.

[70] Ferreira E M, Zhang H, Stoltz B M, Oestreich M (2009). Oxidative Heck-Type Reactions (Fujiwara–Moritani Reactions). The Mizoroki–Heck Reaction.

[71] Rauf W, Brown J M (2013). Chem Commun.

[72] Zhou L, Lu W (2014). Chem – Eur J.

[73] Zoller J, Fabry D C, Ronge M A, Rueping M (2014). Angew Chem, Int Ed.

[74] Fabry D C, Zoller J, Raja S, Rueping M (2014). Angew Chem, Int Ed.

[75] Fabry D C, Ronge M A, Zoller J, Rueping M (2015). Angew Chem, Int Ed.

[76] Choi S, Chatterjee T, Choi W J, You Y, Cho E J (2015). ACS Catal.

[77] Liu K, Zou M, Lei A (2016). J Org Chem.

[78] Kalsi D, Dutta S, Barsu N, Rueping M, Sundararaju B (2018). ACS Catal.

[79] Kim H J, Fabry D C, Mader S, Rueping M (2019). Org Chem Front.

[80] Kalyani D, McMurtrey K B, Neufeldt S R, Sanford M S (2011). J Am Chem Soc.

[81] Kalyani D, Deprez N R, Desai L V, Sanford M S (2005). J Am Chem Soc.

[82] Maestri G, Malacria M, Derat E (2013). Chem Commun.

[83] Neufeldt S R, Sanford M S (2012). Adv Synth Catal.

[84] Jiang J, Zhang W-M, Dai J-J, Xu J, Xu H-J (2017). J Org Chem.

[85] Cismesia M A, Yoon T P (2015). Chem Sci.

[86] Sahoo M K, Midya S P, Landge V G, Balaraman E (2017). Green Chem.

[87] Liang L, Xie M-S, Wang H-X, Niu H-Y, Qu G-R, Guo H-M (2017). J Org Chem.

[88] Czyz M L, Lupton D W, Polyzos A (2017). Chem – Eur J.

[89] Zhou C, Li P, Zhu X, Wang L (2015). Org Lett.

[90] Xu N, Li P, Xie Z, Wang L (2016). Chem – Eur J.

[91] Cambié D, Bottecchia C, Straathof N J W, Hessel V, Noël T (2016). Chem Rev.

[92] Santoro S, Ferlin F, Ackermann L, Vaccaro L (2019). Chem Soc Rev.

[93] Govaerts S, Nyuchev A, Noël T (2020). J Flow Chem.

[94] Sharma U K, Gemoets H P L, Schröder F, Noël T, Van der Eycken E V (2017). ACS Catal.

[95] Manna M K, Bairy G, Jana R (2017). Org Biomol Chem.

[96] McCann S D, Stahl S S (2015). Acc Chem Res.

[97] Almasalma A A, Mejía E (2020). Synthesis.

[98] Chen X, Tan Z, Gui Q, Hu L, Liu J, Wu J, Wang G (2016). Chem – Eur J.

[99] Bai P, Sun S, Li Z, Qiao H, Su X, Yang F, Wu Y, Wu Y (2017). J Org Chem.

[100] Gandeepan P, Koeller J, Korvorapun K, Mohr J, Ackermann L (2019). Angew Chem, Int Ed.

[101] Sagadevan A, Greaney M F (2019). Angew Chem, Int Ed.

[102] Thongpaen J, Manguin R, Dorcet V, Vives T, Duhayon C, Mauduit M, Baslé O (2019). Angew Chem, Int Ed.

[103] Kuhl N, Hopkinson M N, Wencel-Delord J, Glorius F (2012). Angew Chem, Int Ed.

[104] Gauchot V, Sutherland D R, Lee A-L (2017). Chem Sci.

[105] Liang Y-F, Steinbock R, Yang L, Ackermann L (2018). Angew Chem, Int Ed.

[106] Yang F, Koeller J, Ackermann L (2016). Angew Chem, Int Ed.

[107] Gandeepan P, Mo J, Ackermann L (2017). Chem Commun.

[108] Koeller J, Gandeepan P, Ackermann L (2019). Synthesis.

[109] Chinchilla R, Nájera C (2007). Chem Rev.

[110] Wu Y, Huo X, Zhang W (2020). Chem – Eur J.

[111] Karak M, Barbosa L C A, Hargaden G C (2014). RSC Adv.

[112] Chinchilla R, Nájera C (2011). Chem Soc Rev.

[113] Osawa M, Nagai H, Akita M (2007). Dalton Trans.

[114] Tlahuext-Aca A, Hopkinson M N, Sahoo B, Glorius F (2016). Chem Sci.

[115] Jeffrey J L, Terrett J A, MacMillan D W C (2015). Science.

[116] Shaw M H, Shurtleff V W, Terrett J A, Cuthbertson J D, MacMillan D W C (2016). Science.

[117] Twilton J, Christensen M, DiRocco D A, Ruck R T, Davies I W, MacMillan D W C (2018). Angew Chem, Int Ed.

[118] Ahneman D T, Doyle A G (2016). Chem Sci.

[119] Shen Y, Gu Y, Martin R (2018). J Am Chem Soc.

[120] Le C, Liang Y, Evans R W, Li X, MacMillan D W C (2017). Nature.

[121] Joe C L, Doyle A G (2016). Angew Chem, Int Ed.

[122] Deng H-P, Fan X-Z, Chen Z-H, Xu Q-H, Wu J (2017). J Am Chem Soc.

[123] Go S Y, Lee G S, Hong S H (2018). Org Lett.

[124] Huang L, Rueping M (2018). Angew Chem, Int Ed.

[125] Mitsunuma H, Tanabe S, Fuse H, Ohkubo K, Kanai M (2019). Chem Sci.

[126] Zhang X, MacMillan D W C (2017). J Am Chem Soc.

[127] Perry I B, Brewer T F, Sarver P J, Schultz D M, DiRocco D A, MacMillan D W C (2018). Nature.

[128] Sarver P J, Bacauanu V, Schultz D M, DiRocco D A, Lam Y-h, Sherer E C, MacMillan D W C (2020). Nat Chem.

